# Loss‐function mutants of *OsCKX* gene family based on CRISPR‐Cas systems revealed their diversified roles in rice

**DOI:** 10.1002/tpg2.20283

**Published:** 2023-01-20

**Authors:** Xuelian Zheng, Shuting Zhang, Yanling Liang, Rui Zhang, Li Liu, Pengchen Qin, Zhe Zhang, Yan Wang, Jianping Zhou, Xu Tang, Yong Zhang

**Affiliations:** ^1^ Dep. of Biotechnology School of Life Sciences and Technology Center for Informational Biology Univ. of Electronic Science and Technology of China Chengdu 610054 China

## Abstract

Cytokinin (CTK) is an important plant hormone that promotes cell division, controls cell differentiation, and regulates a variety of plant growth and development processes. Cytokinin oxidase/dehydrogenase (CKX) is an irreversible cytokinin‐degrading enzyme that affects plant growth and development by regulating the dynamic balance of CTKs synthesis and degradation. There are presumed 11 members of the CKX gene family in rice (*Oryza sativa* L.), but limited members have been reported. In this study, based on CRISPR‐Cas9 and CRISPR‐Cas12a genome‐editing technology, we established a complete set of *OsCKX1‐OsCKX11* single‐gene mutants, as well as double‐gene and triple‐gene mutants of different *OsCKXs* gene combinations with high similarity. The results revealed that CRISPR‐Cas12a outperformed Cas9 to generate biallelic mutations, multi‐gene mutants, and more diverse genotypes. And then, we found, except the reported *OsCKX2*, *OsCKX4*, *OsCKX9* and *OsCKX11*, *OsCKX5, OsCKX6, OsCKX7*, and *OsCKX8* also had significant effects on agronomic traits such as plant height, panicle size, grain size, and grain number per panicle in rice. In addition, the different loss‐of‐function of the *OsCKX* genes also changed the seed appearance quality and starch composition. Interestingly, by comparing different combinations of multi‐gene mutants, we found significant functional redundancy among *OsCKX* gene members in the same phylogenetic clade. These data collectively reveal the diversified regulating capabilities of *OsCKX* genes in rice, and also provide the valuable reference for further rice molecular breeding.

## Core Ideas

- CRISPR‐Cas12a outperformed CRISPR‐Cas9 to generate multi‐gene mutants.
- There was significant functional redundancy among the *OsCKX* members in the same clade.
- *OsCKX*s affected plant development, seed appearance quality, and starch composition by regulating endogenous cytokinins.
- *OsCKX1/2* and *OsCKX3/8/10* gene clades played key roles in the control of panicle architecture and grain number.
- *OsCKX4/5/9* gene clade regulated the development of roots and plant architecture such as plant height and tillers.

**Abbreviations**
CKX: cytokinin oxidases/dehydrogenase
CTK: cytokinin
PCR: polymerase chain reaction
SL: strigolactone
WT: wild type

## 1 INTRODUCTION

Cytokinin (CTK) is a class of plant hormone that contains adenine derivatives and plays a key role in plant growth and development. Cytokinin has been found to be involved in promoting cell division, regulating cell differentiation, responding to stress and aging, as well as delaying the degradation of protein and chlorophyll (Haberer & Kieber, 2002; Li et al., 2021; Liu et al., 2020; Sakakibara, 2006; Wang et al., 2020b; Werner & Schmulling, 2009; Werner et al., 2001; Wu et al., 2021). Current studies have shown that CTKs are synthesized by the ISOPENTENYL TRANSFERASE (IPT) and LONELY GUY (LOG) enzymes (Avalbaev et al., 2012; Chen et al., 2022; Frebort et al., 2011), while conjugation and destruction of CTKs is carried out through cytokinin oxidases/dehydrogenase (CKX) enzymes or through conjugation to glucose by cytokinin glycosyltransferases (Chen et al., 2020, 2021; Wang et al., 2020b; Werner & Schmulling, 2009; Werner et al., 2006). The CKX is the only enzyme that can irreversibly inactivate CTKs, which belongs to a FAD‐dependent oxidase and affects the growth and development of plants by regulating the dynamic balance of synthesis and degradation of different CTKs (Avalbaev et al., 2012; Dabravolski & Isayenkov, 2021; Werner et al., 2006).

In higher plants, CKXs are encoded by multi‐gene families. Different numbers of *CKX* gene families have been identified or deduced in Arabidopsis, rice (*Oryza sativa* L.), barley (*Hordeum vulgare* L.), wheat (*Triticum aestivum* L.), maize (*Zea mays* L.), cotton (*Gossypium hirsutum* L.), alfalfa (*Medicago sativa* L.), pea (*Pisum sativum* L.), and other plants. (Bilyeu et al., 2001; Chen et al., 2020; Galuszka et al., 2004; Houba‐Herin et al., 1999; Liu et al., 2018, 2013; Mameaux et al., 2012; Morris et al., 1999; Nguyen et al., 2021; Wang et al., 2021; Yang et al., 2003; Zalewski et al., 2014). There are seven members of the Arabidopsis *CKXs* gene family (*AtCKX1‐AtCKX7*). *AtCKXs* genes regulate the development of roots, leaves, and fruits of Arabidopsis, respectively (Bartrina et al., 2011; Kowalska et al., 2010; Werner et al., 2001). There are 13 *CKX* genes in the maize genome (*ZmCKX1‐ZmCKX12, ZmCKX4b*), which have an important impact on maize plant morphology (Wang et al., 2020a; Zalabak et al., 2014). Previous studies have shown that *ZmCKX1‐ZmCKX5* is expressed in the early development of corn grains (Bilyeu et al., 2001; Smehilova et al., 2009). *ZmCKX1* was the first CKX gene cloned and played an important role in regulating corn seed size and grain number (Houba‐Herin et al., 1999; Morris et al., 1999; Schmulling et al., 2003).

There are 11 *CKX* genes (*OsCKX1*‐*OsCKX11*) have been annotated in rice genome (Ashikari et al., 2005; Chen et al., 2020; Tsai et al., 2012). *OsCKX2*, or Gn1a (grain count per grain gene), was the first member of the rice *CKXs* gene family studied extensively (Ashikari et al., 2005). Loss‐of‐function mutant of *OsCKX2* or inhibiting the expression of *OsCKX2* caused an increase in the grains number of rice, which significantly increased the yield (Ashikari et al., 2005; Li et al., 2016; Yeh et al., 2015). Recently, some members of the *OsCKX* gene family were characterized. *OsCKX4* was shown to mainly express in roots and leaves and regulated the development of rice crown roots. Reducing *OsCKX4* expression or knocking out *OsCKX4* resulted in CTK accumulation in rice, inhibiting root growth and causing significantly shorter roots (Gao et al., 2014; Rong et al., 2022). In addition, the *OsCKX9* gene was found as a downstream response gene of the strigolactone (SL) signaling pathway, induced by SL analogues, but not the CTKs. The CRISPR‐Cas9 knockout mutants of *OsCKX9* exhibited significantly different phenotypes from that of the wild type (WT), such as shorter plant height, increased tiller number, and shorter panicles (Duan et al., 2019; Rong et al., 2022). The double mutant of *OsCKX4* and *OsCKX9* led to significant changes in agronomic traits of rice such as increased tiller number, smaller panicle size, increased effective panicle number, and significant decrease in the grains number per panicle (Rong et al., 2022). More recently, it was reported that *OsCKX11* was associated with senescence in rice leaves. And knocking out *OsCKX11* resulted in an increase of the panicles number and the tiller number (Rong et al., 2022; Zhang et al., 2021a).

**Core Ideas**

- CRISPR‐Cas12a outperformed CRISPR‐Cas9 to generate multi‐gene mutants.
- There was significant functional redundancy among the OsCKX members in the same clade.
- OsCKXs affected plant development, seed appearance quality, and starch composition by regulating endogenous cytokinins.
- *OsCKX1/2* and *OsCKX3/8/10* gene clades played key roles in the control of panicle architecture and grain number.
- *OsCKX4/5/9* gene clade regulated the development of roots and plant architecture such as plant height and tillers.

In this study, we sought to characterize the roles of all 11 *OsCKX* genes using the multiplexed CRISPR‐Cas gene‐editing technology. We first knocked out *OsCKXs* gene family members in Nipponbare and created a complete *OsCKX1‐OsCKX11* single‐gene mutant library and multi‐gene mutant materials by CRISPR‐Cas9 and CRISPR‐Cas12a genome‐editing technique. We then evaluated the phenotypes of *OsCKXs* single‐gene mutants and different combinations of multi‐gene mutants. This would help us understand the function of the individual gene member of the *OsCKXs* gene family, as well as the relationships and interaction patterns of the family members. Our findings can provide materials support for further study of the biological function of the *OsCKX* gene family. It can also provide a basis for rice‐breeding improvement.

## 2 MATERIALS AND METHODS

### 2.1 Plant materials and growth conditions

The rice cultivar Nipponbare (*O. sativa* L. *japonica*) was used as the WT control in present study. All mutants were created based on Nipponbare. The T2 generation of homozygote mutants without transgene were used for phenotype and molecular characterization. The plants were grown in climate chambers under long‐day conditions (16 h light at 30 °C and 8 h dark at 24 °C) and in greenhouse for experiment.

### 2.2 Construction of the vectors

We used Cas9 expression backbone vector pGEL026 (pZmUbi::Cas9::HspT‐pOsU6::sgRNA::pT) (Ren et al., 2019; Zhou et al., 2019) for construction all the single‐gene‐knockout vectors, from pCKX05 to pCKX15 (Supplemental Table S2). The different recombinant fragments OsU6 promoter‐OsCKX‐sgRNA‐scaffolds‐terminator were cloned into pGEL026 using fusion polymerase chain reaction (PCR) followed by ligation. All the primers were listed in Supplemental Table S3. For Cas12a vectors from pCKX16 to pCKX26 (Supplemental Table S2), the backbone pGEL632 (pZmUbi::Cas12a::HspT‐pOsUbi::crRNA::pT) (Tang et al., 2017, 2016) was used. The different expression cassettes of OsUbi promoter‐OsCKX‐crRNA scaffolds‐terminator were cloned into pTX377 using fusion PCR followed by ligation. All the primers are listed in Supplemental Table S3.

For multi‐gene knockout vectors pWY27, pWY28, pWY29, and pWY30 (Supplemental Table S2), the OsUbi promoter‐three crRNA fragments‐XX terminator fusion PCR fragment was cloned to the backbone vector pGEL632. The PCR fragment was cut by BamHI and SpeI, then cloned into digested pGEL632. All the oligos are listed in Supplemental Table S3.

### 2.3 Rice stable transformation

*Agrobacterium tumefaciens* mediated rice stable transformation was carried out according to a previously published protocol (Ren et al., 2021; Tang et al., 2017, 2016; Wang et al., 2019; Zhou et al., 2019).

### 2.4 Detection of targeted mutations in rice materials

Total genomic DNA was extracted using the CTAB method. Specific primers (Supplemental Table S3) were designed according to the vector, and the plant genomic DNA was used as a template for PCR amplification to detect the existence of the vector in the plant. The amplified products were analyzed with 1% agarose gel. Genomic regions of targeted sites were amplified with specific primers for the detection of chromosomal deletions (Supplemental Table S3). The PCR products were analyzed by polymerase chain reaction‐single strand conformation polymorphism (PCR‐SSCP) (Zheng et al., 2016). All the T0 and T1 mutant lines were further genotyped by Sanger sequencing.

### 2.5 Investigation on agronomic characters of rice

The T1 generations originated from T0 mutation lines were used to test agronomic traits. The rice yield‐related traits were measured according to a previous method (Zhou et al., 2019). At least three individual plants were used for data collection of each genotype. Each sample was tested for three times.

### 2.6 Determination of plant hormone and starch content

In this experiment, the determination of plant hormone, total starch content and amylose content were entrusted to Shanghai sanshu Biotechnology Company.

### 2.7 Statistical analysis

All statistical analyses were performed with GraphPad Prism 8.0.1.244. Error bars represent standard deviations of at least three biological replicates. Data are means ± SD. Student's *t* test was used for pairwise comparison. Asterisks indicated significant differences (*p < .05, **p < .01, *** *p* < .001) between two treatments.

## 3 RESULTS

### 3.1 Characterization of the *OsCKX* gene family and creation of the *OsCKXs* single‐gene mutants

**FIGURE 1:**
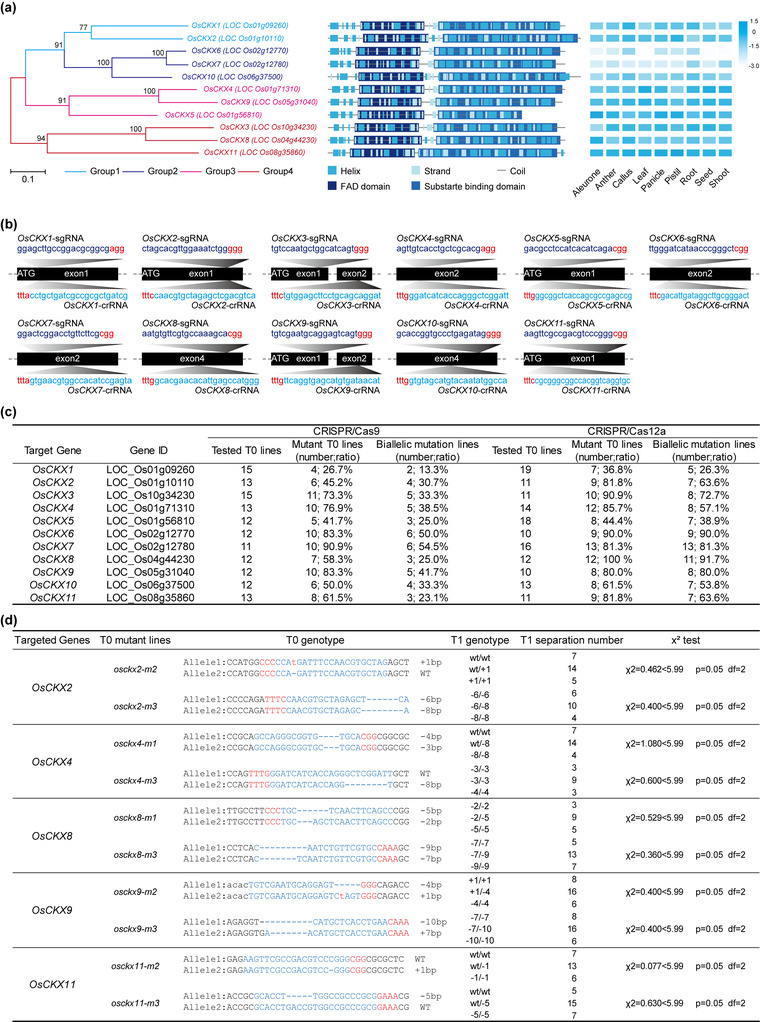
Creation of *OsCKXs* single‐gene mutants in rice with CRISPR‐Cas9 and CRISPR‐Cas12a. (a) Phylogenetic tree and predicted protein secondary structure of 11 rice *OsCKX* genes. The tree was constructed using MEGA7. The FAD‐binding domain and cytokinin‐binding domain were showed in green and blue. The expression pattern of 11 *OsCKX* genes in various rice tissues according to Rice Expression Database (http://expression.ic4r.org/). The expression pattern was based on log10 (FPKM) values. (b) The sgRNA and crRNA were designed to target the different *OsCKX* genes based on CRISPR‐Cas9 and CRISPR‐Cas12a. The PAM sequence was highlighted in red, the protospacer sequence was highlighted in green and blue respectively. (c) Summary of mutation frequencies of CRISPR‐Cas9 and CRISPR‐Cas12a system at different *OsCKXs* locus in T0 rice transgenic lines. (d) Heredity test of the mutations at partial *OsCKX* locus in the T1 generation

The CKXs were a multi‐gene family in plants. The information of 11 gene members of rice *OsCKXs* gene family was obtained and identified from RICE DATA CENTER databases (https://ricedata.cn/) (Supplemental Figure S1a). Phylogenetic analysis showed that the *OsCKXs* genes grouped into four distinct clades. Each clade consisted of two to three genes: *OsCKX1* and *OsCKX2*; *OsCKX6, OsCKX7* and *OsCKX10*; *OsCKX4*, *OsCKX5* and *OsCKX9*; *OsCKX3, OsCKX8* and *OsCKX11* (Figure 1a). Protein motif analysis showed that most of the motifs have a consistent pattern corresponding to the phylogenetic clustering. Similar to the Arabidopsis CKX proteins, *OsCKXs* had a conserved FAD domain and a C‐terminal substrate binding domain (Figure 1a; Supplemental Figure S1b). Based on the analysis of the expression patterns of individual *OsCKXs* gene family members (http://expression.ic4r.org/), the *OsCKXs* genes demonstrated clear tissue specificity in nine different tissues including roots, leaves, spike (panicle) and grain (seed) (Figure 1a). *OsCKX11* was the most highly expressed *OsCKX* gene family member and constitutively expressed in all examined tissues. On the contrary, *OsCKX6* and *OsCKX7* were expressed at very low levels in almost all the tissues. *OsCKX2* was similar to *OsCKX11* and expressed in the highest amount in pistil but was expressed lower than *OsCKX11* in roots. *OsCKX1, OsCKX3*, and *OsCKX4* were expressed highly in roots, and *OsCKX4* was also highly expressed in leaves. The diverse expression pattern of the 11 *OsCKX* gene family members indicated subfunctionalization of the individual gene families.

To obtain loss‐of‐function mutants of 11 *OsCKXs* gene family members, we used CRISPR‐Cas9 (Ren et al., 2021, 2019; Wang et al., 2019) and CRISPR‐Cas12a (Tang et al., 2017; Zhang et al., 2021a) genome‐editing systems to introduce targeted mutations in each single gene, respectively. From *OsCKX1* to *OsCKX11*, the corresponding guide RNA sequences (sgRNA for Cas9 system, and crRNA for Cas12a system) were designed as our previously reported strategies (Figure 1b). We obtained the 11 *OsCKXs* single‐gene knockout mutants with CRISPR‐Cas9 and CRISPR‐Cas12a systems, respectively. The target editing efficiency of the Cas9 system ranged from 26.7 to 90.0%, and the efficiency at 8 out of 11 sites were more than 50%. The editing efficiency of the Cas12a system ranged from 36.8% (OsCKX5‐crRNA) to 100% (OsCKX8‐crRNA), and the efficiency was above 60% at nine sites. In addition, the biallelic mutation efficiency of Cas9 system was up to 54.5%, 2 out of 11 sites was above 50%. In Cas12a system, the biallelic mutation efficiency of the 8 out of 11 were above 50%, and the highest was 91.7% (OsCKX8‐crRNA) (Figure 1c). The results showed that the efficiency and stability of the CRISPR‐Cas12a system were higher than that of the CRISPR‐Cas9 system. This was consistent with previous reports (Tang et al., 2017; Wu et al., 2022). We performed genotype analysis on partial mutants, and the results showed that although Cas9 system also had large fragment deletions, most of the mutations of Cas9 system were 1–3 bp InDel (insertions and deletions), especially 1 bp InDel. Although Cas12a system was mostly more than 5 bp deletions, and the mutant genotypes were more abundant and diverse in Cas12a system (Figure 1d; Supplemental Figure S2). We further performed genotype identification and χ^2^‐square detection of *OsCKXs* T1 mutant materials. The results showed that the genotype of the T1 isolated materials, both the Cas9 system and the Cas12a system, conformed to the Mendelian segregation, and could be stably inherited (Figure 1d; Supplemental Table S1). It was further demonstrated that the Cas12a system was just as effective, or a better genome‐editing tool than Cas9 system, and the mutant genotype was more abundant.

### 3.2 *OsCKX* single‐gene mutants have different effects on rice growth and development

**FIGURE 2:**
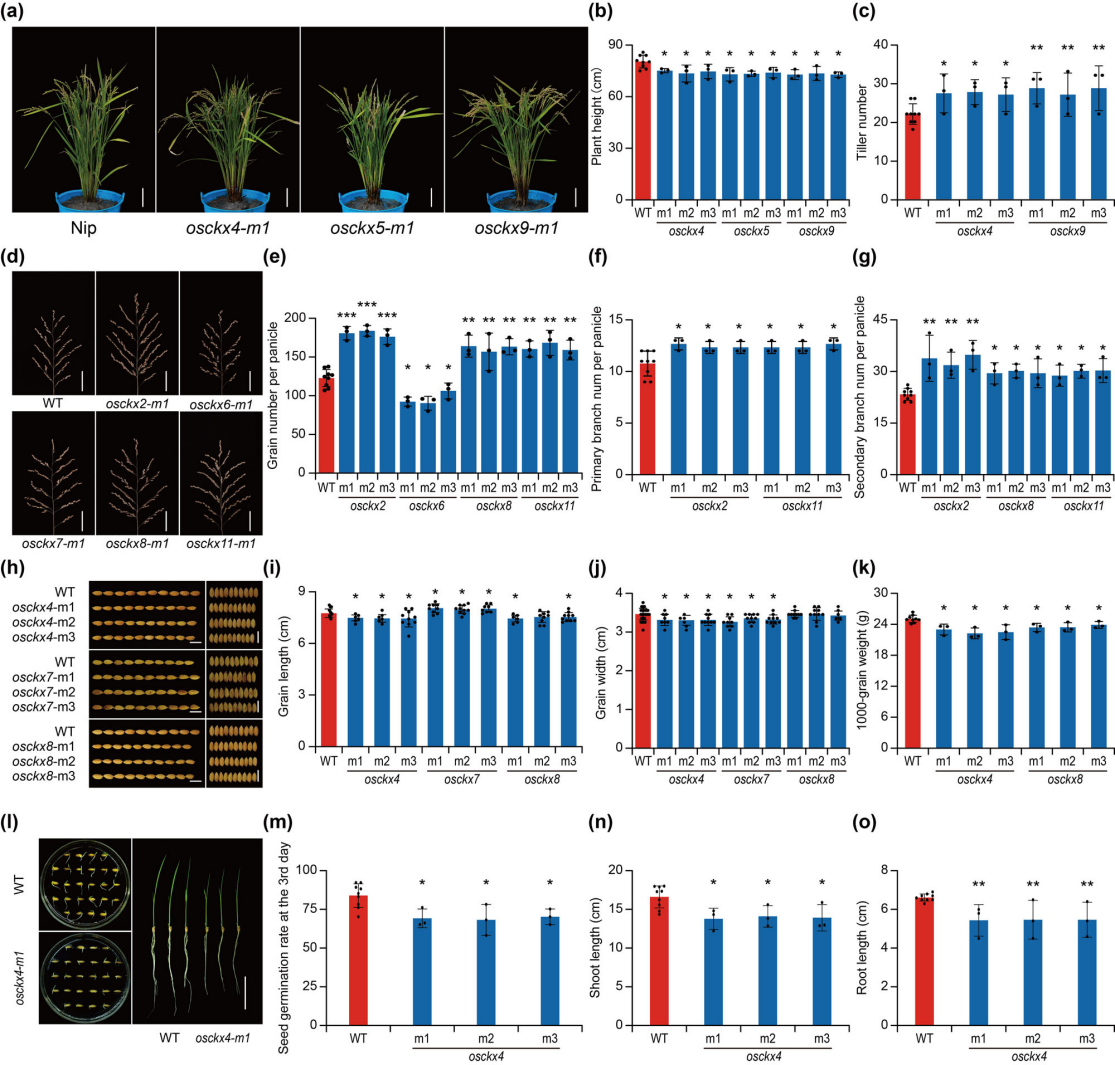
Phenotypic analysis of different *OsCKXs* single‐gene mutants. (a) The plant architecture of *OsCKX4, OsCKX5, OsCKX9* mutants and wild type (WT) at the ripening stage. Bar = 10 cm. (b) Plant height of the *OsCKX4, OsCKX5, OsCKX9* mutants and WT. (c) Tiller number of the *OsCKX4, OsCKX9* mutants and WT. (d) Panicle type of *OsCKX2, OsCKX6, OsCKX7, OsCKX8, OsCKX11* mutants and WT. Bar = 5 cm. Grain number per panicle (e), primary branch number per panicle (f) and secondary branch number per panicle (g) of different *OsCKX* mutants and WT. (h) Grain size of *OsCKX4, OsCKX7, OsCKX8* mutants and WT. Bar = 1 cm. Grain length (i), grain width (j) and 1,000‐grain weight (k) of different *OsCKX* mutants and WT. (l) Seed germination (bar = 1 cm) and seedling growth (bar = 5 cm) of *OsCKX4* mutant and WT. Seed germination rate (*n* = 30 * 3) (m), shoot length (*n* = 10 * 3) (*n*) and root length (*n* = 10 *3) (o) of *OsCKX4* mutant and WT. Error bars represent standard deviations of three biological replicates. Data are means ± SD. Statistical significance is indicated by asterisks. *Indicative of a *p* value < .05 by the Student's *t* test. **Indicative of a *p* value < .01 by the Student's *t* test. ***Indicative of a *p* value < .001 by the Student's *t* test

We selected three vector‐free biallelic mutants with different genotypes for each gene to propagate. And analysis of agronomic traits of mutant materials planted in the field to study the functions of different single *OsCKXs*. We found that the knockout of specific *OsCKXs* resulted in significantly lower plant height than WT in *osckx4*, *osckx5*, and *osckx9* single‐gene mutants (Figure 2a and 2b; Supplemental Figure S4a), as well as the significantly increasing of tiller numbers in *osckx4* and *osckx9* mutants (Figure 2c; Supplemental Figure S4b), which was consistent with previous reports (Duan et al., 2019; Gao et al., 2014; Rong et al., 2022). Panicle size at the yellow ripening stage showed a significant decrease of panicle length in *osckx6*, and an increase in *osckx7* (Figure 2d; Supplemental Figure S5b). Consistent with Ashikari's reports (Ashikari et al., 2005), the *osckx2* knockout mutant showed a significant increase of the grain number per panicle (GNP). Compared with the 125 GNP of the WT, the GNP of *osckx2* reached about 180 (Figure 2e). We also observed the GNP of *osckx8* and *osckx11* mutants increased significantly to about 165. Although the GNP of *osckx6* mutant was reduced to about 95 (Figure 2e; Supplemental Figure S5a). The investigation of the primary branch number per panicle showed that there was no difference in individual *osckxs* mutants, except for an increase in *osckx2* and *osckx11* (Figure 2f; Supplemental Figure S6a) The results of the secondary branch number per panicle showed significant increases in the *osckx2*, *osckx8*, and *osckx11* mutants (Figure 2g; Supplemental Figure S6b). These results inferred that the increase of seed density caused by the increase of secondary branch number per panicle was the main reason for the increase of the GNP in *osckx2*, *osckx8*, and *osckx11* mutants. Notably, the seed setting rates in *osckx4*, *osckx7*, and *osckx11* were significantly lower than that of WT (Supplemental Figure S4c), which might result in no significant increase of the yield per plant in *osckx11*.

For the effects on grain size, the grain lengths of *osckx4* and *osckx8* were shorter than that of WT, whereas the *osckx7* was longer (Figure 2h and 2i; Supplemental Figure S8b). The grain widths of *osckx4* and *osckx7* were thinner than that of WT, and there was no significant difference in *osckx8* (Figure 2h and 2j; Supplemental Figure S8c). Although the grain size of *osckx7* was thinner and longer than WT, the 1,000‐grain weight was around 25.5 g and was not significantly affected. The smaller grain size of *osckx4* led to a reduction of 1,000‐grain weight to 22.5 g, which could result in a decrease of the yield per plant. The grain size of *osckx8* became smaller, which also caused the decrease of the 1,000‐grain weight to around 23.8 g (Figure 2k; Supplemental Figure S8a). However, due to the increase of GNP in *osckx8*, the yield per plant of *osckx8* might not change significantly. There were no differences between the other *OsCKXs* single‐gene mutants and the WT (Supplemental Figure S7 and S8).

The seed germination rate and seedling growth were further investigated of all the *osckxs* mutants. The seed germination rate at the 3rd day of *CKX4* was only about 60%, which was significantly lower than that of WT about 85% (Figure 2l and 2m ). Interestingly, there was no significant difference in seed germination rate at the 7th day (data not shown). However, the shoot length and root length of *osckx4* after 7 d of germination were significantly shorter than that of WT (Figure 2l, 2n, and 2o). These results were consistent with previous reports that *OsCKX4* gene mainly regulated the development of rice roots. Except for *osckx4*, none of the other *OsCKXs* single‐gene mutants exhibited differences from the WT (Supplemental Figure S9).

### 3.3 Generation of *OsCKXs* multi‐gene mutants and phenotypic analysis

**FIGURE 3:**
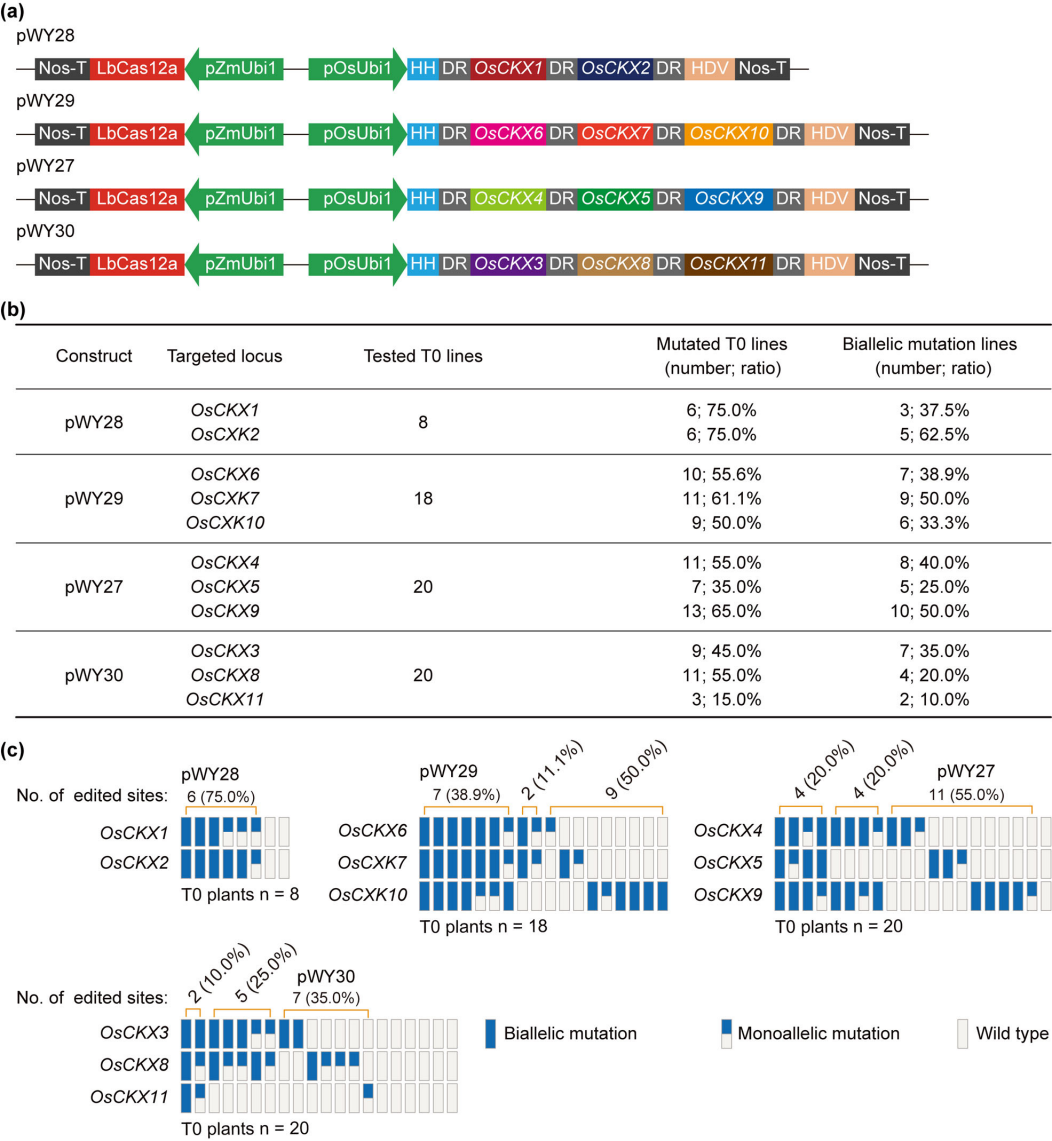
Creation of *OsCKXs* multiple‐gene mutants in rice with CRISPR‐Cas12a. (a) Schematics of the multiplexed CRISPR‐Cas12a expression constructs (based on pGEL632 backbone) for targeted different *OsCKXs* genes. The Cas12a gene was expressed under a maize ubiquitin 1 promoter (pZmUbi1) and the crRNAs were expressed under a rice ubiquitin 1 promoter (pOsUbi). (b) A summary table of mutation frequencies in T0 rice transgenic lines at different target site by different construct. (c) A schematic presentation of the genotyping results for all T0 lines categorized as biallelic mutation, monoallelic mutation, and wildtype (WT)

In order to study the function and relationship between *OsCKXs* gene family members, we combined the more closely related genes according to the phylogenetic tree to obtain four clades, of multi‐gene knockout mutants: *OsCKX1/2* (*OsCKX1* and *OsCKX2*), *OsCKX3/8/11* (*OsCKX3, OsCKX8* and *OsCKX11)*, *OsCKX4/5/9* (*OsCKX4, OsCKX5* and *OsCKX9*), and *OsCKX6/7/10* (*OsCKX6, OsCKX7* and *OsCKX10*) (Figures 1a and 3a). The multi‐gene knockout vectors based on the Cas12a system showed high co‐knockout efficiency. Except for OsCKX11‐crRNA, the knockout efficiency of all crRNAs was more than 50% (Figure 3b). We obtained seven different double‐gene mutants and triple‐gene mutants, *osckx1/2, osckx3/8, osckx3/8/11, osckx4/9, osckx4/5/9, osckx6/7*, and *osckx6/7/10*, completely covering four distinct clades of *OsCKXs* gene family (Figure 3c; Supplemental Figure S3).

**FIGURE 4:**
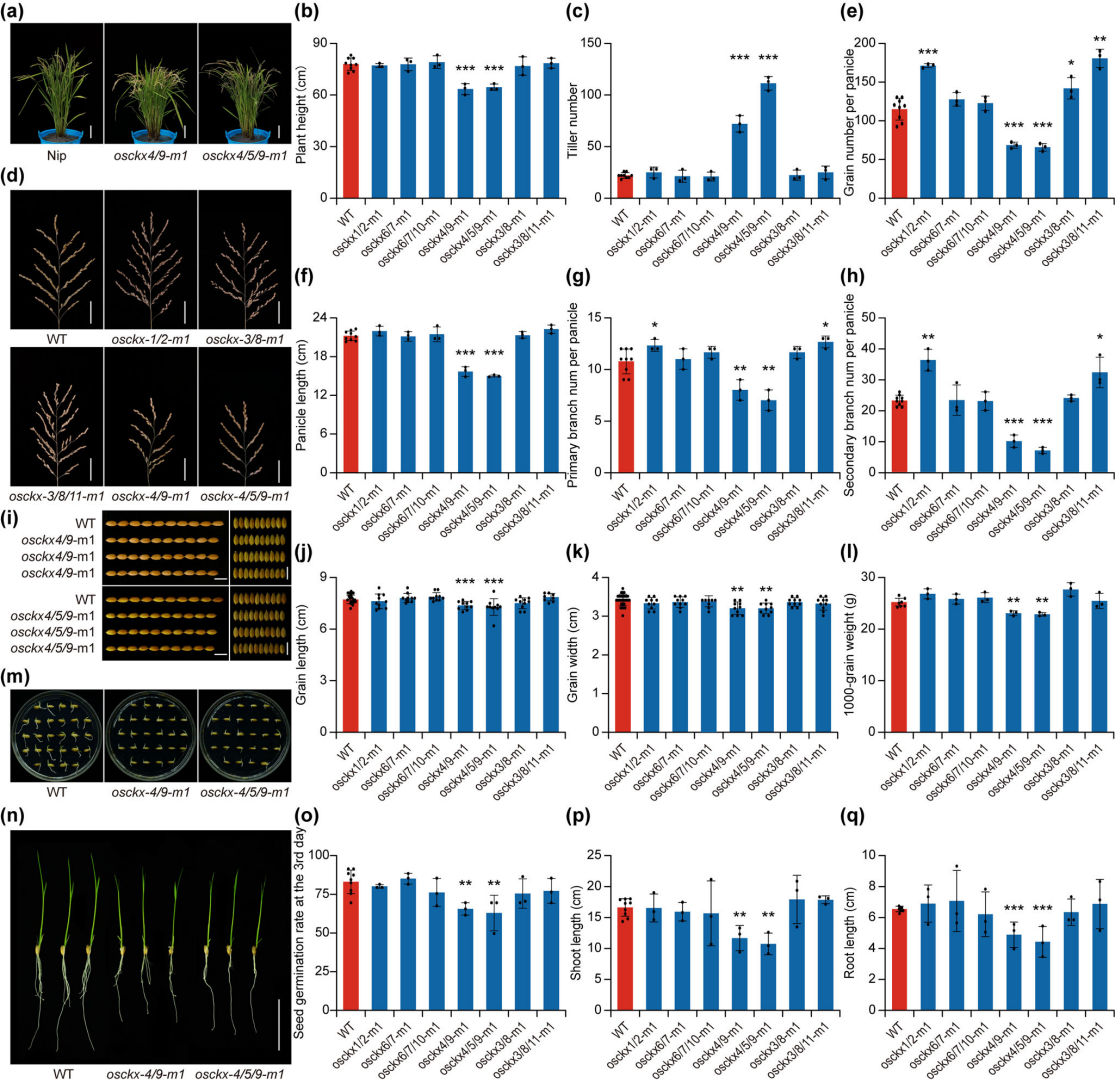
Phenotypic analysis of different *OsCKXs* multiple‐gene mutants. (a) The plant architecture of *OsCKX4/9* double mutant*, OsCKX4/5/9* triple mutant and wild type (WT) at the ripening stage. Bar = 10 cm. Plant height (b) and tiller number (c) of all the seven types of *OsCKXs* multiple‐gene mutants and WT. (d) Panicle type of different *OsCKXs* multiple‐gene mutants and WT. Bar = 5 cm. Grain number per panicle (e) grain length (f) primary branch number per panicle (g) and secondary branch number per panicle (h) of all the *OsCKXs* multiple‐gene mutants and WT. (i) Grain size of *OsCKX4/9* double mutant*, OsCKX4/5/9* triple mutant and WT. Bar = 1 cm. Grain length (j), grain width (k) and 1,000‐grain weight (l) of all the *OsCKXs* multiple‐gene mutants and WT. Seed germination (bar = 1 cm) (m) and seedling growth (bar = 5 cm) (n) of *OsCKX4/9* double mutant*, OsCKX4/5/9* triple mutant and WT. Seed germination rate (*n* = 30 * 3) (o) shoot length (*n* = 10 * 3) (p) and root length (*n* = 10 * 3) (q) of all the *OsCKXs* multiple‐gene mutants and WT. Error bars represent standard deviations of three biological replicates. Data are means ± SD. Statistical significance is indicated by asterisks. *Indicative of a *p* value < .05 by the Student's *t* test. **Indicative of a *p* value < .01 by the Student's *t* test. ***Indicative of a *p* value < .001 by the Student's *t* test

Agronomic traits were investigated for all the multi‐gene mutants. The *osckx4/9* double mutant and *osckx4/5/9* triple mutant were significantly affected in plant height and tiller number. Compared with WT, the plant height of *osckx4/9* and *osckx4/5/9* mutants was reduced by 20% to about 65 cm (Figure 4a and 4b; Supplemental Figure S4a). And the tiller numbers of *osckx4/9* and *osckx4/5/9* mutants were increased by three to five times, respectively, to a maximum of 110 (Figure 4a and 4c; Supplemental Figure S4b). In addition, whereas reduced tiller number in *osckx2* or increased tiller number in *osckx11* were not observed in our single‐gene mutants or related *osckx1/2* and *osckx3/8/11* multi‐gene mutants (Rong et al., 2022) (Supplemental Figure S4b). The discrepancies between our results and previous research might be attributed to different cultivated conditions and environmental impact.

The panicle length of *osckx4/9* and *osckx4/5/9* mutants was significantly shorter than that of WT. No significant changes were observed in other multi‐gene mutants (Figure 4d and 4f; Supplemental Figure S5d). The investigation of the GNP in the multi‐gene mutants showed that there was a remarkable increase in *osckx1/2* and *osckx3/8/11* mutants. Although the GNP was significantly decreased in *osckx4/9* and *osckx4/5/9*, slightly increased in *oscks3/8*, and no differences in *osckx6/7* and *osckx6/7/10* mutants (Figure 4e; Supplemental Figure S5c). Interestingly, the changes of primary and secondary branch number per panicle were almost identical to that of the GNP. The primary and secondary branch number per panicle was increased significantly in *osckx1/2* and *osckx3/8/11* mutants, while decreased significantly in *osckx4/9* and *osckx4/5/9*. And there were no differences in *osckx3/8, osckx6/7*, and *osckx6/7/10* mutants (Figure 4g and 4h; Supplemental Figure S6). With the significant changes in the branches and panicles, the seed setting rates of *osckx3/8/11*, *osckx4/9*, and *osckx4/5/9* were decreased. However, the seed setting rates of *osckx1/2* was not affected (Supplemental Figure S4c).

The grain size (grain length and grain width) of *osckx4/9* double mutant and *osckx4/5/9* triple mutant was significantly smaller than that of the wild type (Figure 4i, 4j and 4k), and the 1,000‐grain weight was lower than that of the WT, too (Figure 4l). No significant changes were observed in other multi‐gene mutants (Supplemental Figure S7 and S8). Similar to the results of the *osckx4* single‐gene mutant, the seed germination rate at the 3rd day of *osckx4/9* and *osckx4/5/9* mutants was lower than that of WT (Figure 4m and 4o; Supplemental Figure S9a). And the shoot length and root length of *osckx4/9* and *osckx4/5/9* mutants was also significantly shorter, too (Figure 4n, 4p and 4q; Supplemental Figure S9b and S9c).

### 3.4 *OsCKXs* genes affected the appearance quality and starch composition of the grain

**FIGURE 5:**
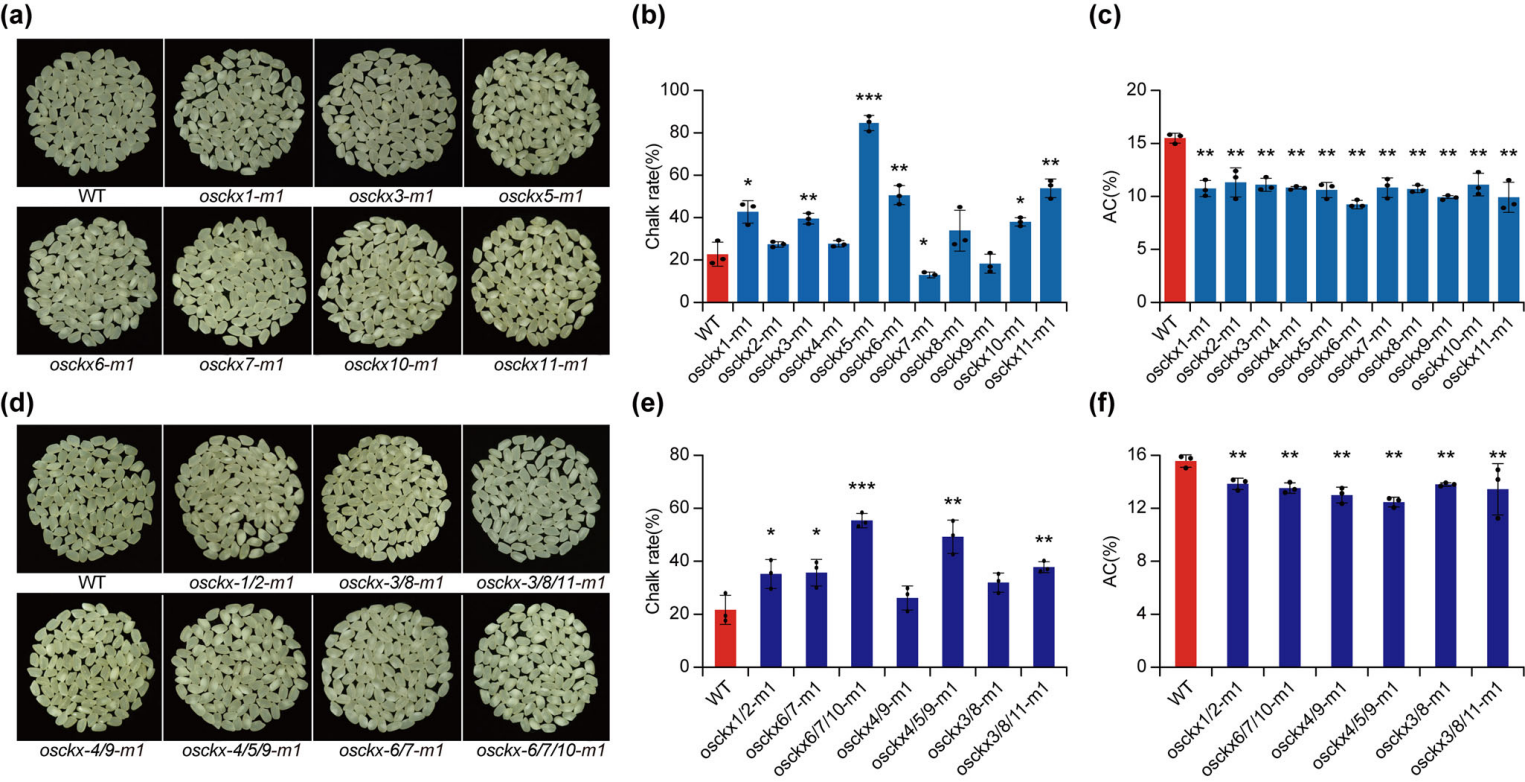
The seed appearance and starch composition of *OsCKXs* single‐gene mutants, multiple‐gene mutants and wild type (WT). (a) The seeds appearance of partial *OsCKXs* single‐gene mutants and WT. Bar = 1 cm. Chalk rate (b) and amylose content (c) of all the *OsCKXs* single‐gene mutants and WT. (d) The seeds appearance of partial *OsCKXs* multiple‐gene mutants and WT. Bar = 1 cm. Chalk rate (e) and amylose content (f) of all the *OsCKXs* multiple‐gene mutants and WT

In addition to yield‐related traits, we also found that knocking out of *OsCKXs* genes had a wide range of effects on the quality of rice grains. Among the 11 single‐gene mutants, the chalky grain rate was slightly decreased in *osckx7*, and increased significantly in *osckx1, osckx3, osckx5, osckx6, osckx10*, and *osckx11* (Figure 5a and 5b). Among them, *osckx5* had the highest chalk grain rate of more than 80% (Figure 5b). The results of multi‐gene mutants showed that the chalk grain rates of *osckx1/2, osckx3/8/11, osckx4/5/9, osckx6/7*, and *osckx6/7/10* were significantly increased (Figure 5d and 5e). All the other single‐gene mutants and multi‐gene mutants were no different from the WT (Supplemental Figure S10a). We further analyzed the total starch content and amylose content of all mutant seeds. The results showed that the total starch content of both single‐gene and multi‐gene mutants remained unchanged (Supplemental Figure S10b), whereas the amylose content decreased to varying degrees compared with WT (Figure 5c and 5f). This might be related to the prolonged growth period of the mutants and the immaturity caused by insufficient grouting.

### 3.5 Changes of endogenous cytokinin levels in *OsCKXs* gene mutants

**FIGURE 6:**
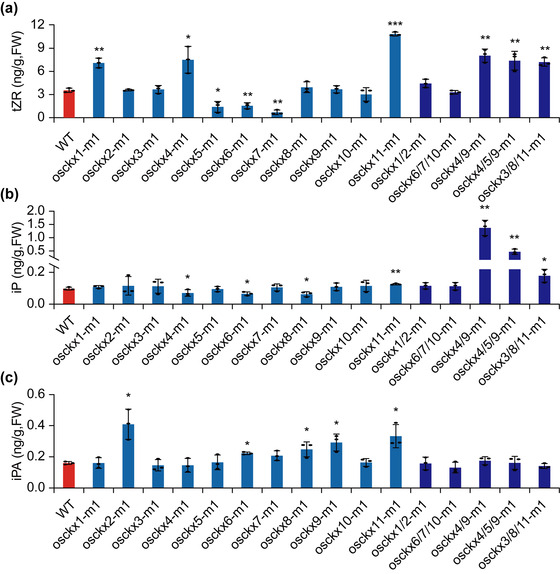
The endogenous CTKs contents of *OsCKXs* single‐gene mutants, multiple‐gene mutants and wild type (WT). (a) Trans‐Zeatin‐riboside (tZR) content of all the *OsCKXs* single‐gene mutants, multiple‐gene mutants and WT. (b) Isopentenyl adenine (iP) content of all the *OsCKXs* single‐gene mutants, multiple‐gene mutants and WT. (c) Isopentenyl adenosine (iPA) content of all the *OsCKXs* single‐gene mutants, multiple‐gene mutants and WT

We detected CTKs contents of 40 d leaves in all *OsCKXs* gene mutants. Trans‐zeatin (tZ) was not detected in all samples, including WT. Trans‐Zeatin‐riboside (tZR) content was increased by 2‐2.5 times in *osckx1*, *osckx4*, *osckx11* single‐gene mutants, and *osckx3/8/11, osckx4/9*, and *osckx4/5/9* multi‐gene mutants. tZR declined in *osckx5, osckx6*, and *osckx7* mutants (Figure 6a). N6‐(Δ2‐Isopentenyl) adenine (iP) content was decreased in *osckx6* and *osckx8* mutants, and significantly increased in *osckx11, osckx3/8/11, osckx4/9*, and *osckx4/5/9*. Especially in *osckx4/9*, iP content remarkably increased by more than 10 times (Figure 6b). N6‐(Δ2‐Isopentenyl) adenosine (iPA) content was increased in *osckx2, osckx6, osckx8, osckx9*, and *osckx11* mutants (Figure 6c). These results showed that the *OsCKXs* were involved in regulating the degradation of tZR and iP in rice leaves. Knocking out different *OsCKXs* reduced the activity of CKX and resulted in the elevation of different CTKs.

## 4 DISCUSSION

### 4.1 CRISPR‐Cas12a outperformed CRISPR‐Cas9 to generate multi‐gene mutants

In recent years, CRISPR‐Cas9 system had been increasingly used in editing the CKX genes and phenomenal results had been achieved (Duan et al., 2019; Li et al., 2016; Mahto et al., 2022; Mandal et al., 2022; Rong et al., 2022; Zhang et al., 2021a). In our research, based on the homology analysis of the members of the *OsCKX* gene family, we used CRISPR‐Cas9 and CRISPR‐Cas12a systems to create a complete library of *OsCKX1∼OsCKX11* single‐gene mutants, as well as double‐gene and triple‐gene mutants. The results revealed that two CRISPR‐Cas systems both showed clear editing activities for different *OsCKXs* gene family members and successfully generated loss‐function mutants, which could be stably inherited. In the detection of T0 generation mutation, it was found that the two CRISPR‐Cas systems had different mutation efficiencies at different sites (Figure 1c). We considered that this was related to complex factors such as chromatin accessibility of the target sites, GC content of target genes, loss‐function, or lethality by gene mutation. CRISPR‐Cas12a recognized a PAM sequence of 5′‐TTTV‐3′ and cleaved at the distal end of the PAM, typically producing a large fragment deletion of 6–13 bp. CRISPR‐Cas12a required only crRNA to guide the Cas12a protein to achieve target site knockout, and the system could self‐process crRNA precursors, suitable for multi‐gene co‐editing. LbCas12a had been shown to achieve high‐efficiency genome editing and mutation in rice (Tang et al., 2017; Zhang et al., 2021b). Our study also demonstrated that CRISPR‐Cas12a had higher biallelic mutant efficiency, more diverse InDel genotype, and more stable editing efficiency, which was more suitable generate multi‐gene mutants.

### 4.2 *OsCKX1/2* and *OsCKX3/8/10* gene clades played key roles in the control of panicle architecture and grain number

The CKX had been reported to affect the development of plant inflorescences in several plants (Chen et al., 2020; Schwarz et al., 2020; Shoaib et al., 2020; Szala et al., 2020). In Arabidopsis, overexpression of the *AtCKX1* and *AtCKX3* genes reduced the inflorescence on each stem of the transgenic plant, as well the ability of the apical inflorescence meristem to form new flower primordia (Werner et al., 2003, 2001). Manipulation of CKX in cereals showed clear impacts on yield (Chen et al., 2020; Dash & Rai, 2022; Holubová et al., 2018; Li et al., 2018; Mandal et al., 2022; Ogonowska et al., 2019). In wheat, down‐regulation of the expression of *TaCKX2.4* by RNAi increased the number of grains per panicle (Li et al., 2018). Similarly, silencing the *HvCKX1* gene expression in barley reduced CKX protein activity and increased grain yield (Zalewski et al., 2010, 2014). Unlike wheat and barley, the rice inflorescence was a panicle. The rice yield was determined by 1,000‐grain weight, GNP and the tiller number (one panicle per tiller) (Chen et al., 2020). The report of Ashikari firstly identified the null mutation of *OsCKX2* increased grain number remarkably in line 5150, a high‐yielding Chinese variety (Ashikari et al., 2005). More recently, researches showed that the gene‐edited mutants of *OsCKX2* in rice increased the plant height, panicle size, spikelet per panicle, and GNP significantly (Li et al., 2016, 2021; Rong et al., 2022; Tu et al., 2022; Yan et al., 2022; Zhou et al., 2019). Moreover, Rong reported that the *osckx1/2* double mutant of Nipponbare showed a significant increase in the number of spikelets per panicle and 1,000‐grain weight, whereas the fewer tillers, less panicle number, lower seed setting rate (Rong et al., 2022). Besides *OsCKX2*, Zhang also characterized the *osckx11* mutants produced more primary branches, higher tiller numbers, increased GNP and total grain number per plant, and had lower fertility, lower 1,000‐grain weight, and smaller seed size (width) (Zhang et al., 2021a).

Our present study showed similar results of GNP in *osckx2* single‐gene mutant. Knocking out *OsCKX2* in Nipponbare caused a significant increase in GNP, and the grain number increase 40−50% compared with the WT (Figure 2e). The specific increase in GNP was accompanied by an increase in the primary and secondary branch number per panicle. There were no significant differences observed in plant height, tiller number, seed setting rate, 1,000‐grain weight, grain length and width, seed germination, seedling growth and other traits. Similar results of dramatically increased GNP in *osckx8* and *osckx11* single‐gene mutants were also observed in our study (Figure 2e). In *osckx11* mutant, in addition to increasing GNP and panicle branches, the seed setting rate was greatly reduced (Supplemental Figure S4c). This was different from the phenotype of *osckx2* mutant, but similar to previous reports (Rong et al., 2022). There was no investigation about the *OsCKX8* affected GNP before. In our present study, knocking out of *OsCKX8* synchronously caused the shorter grain length and less 1,000‐grain weight, in addition to increasing panicle number and secondary panicle branch number (Figure 2i and 2k).

Moreover, *OsCKX1* and *OsCKX2* were grouped into the same clade. The investigation of *osckx1/2* double mutant showed that the GNP also increased remarkably by 40∼50%. The primary branch number and the secondary branch number of panicle had also increased significantly. There were no significant changes in plant height, tiller number, panicle length, seed setting rate, 1,000‐grain weight, and grain size (grain length and width) (Figure 4). These results were exactly consistent with the phenotypes of the *osckx2* single‐gene mutant, which suggested that *OsCKX2* was the main gene that played a major role in *OsCKX1* and *OsCKX2* clade.

*OsCKX3, OsCKX8*, and *OsCKX11* were grouped into the same clade too. The *osckx3/8* double mutant and *osckx3/8/11* triple mutant also presented significant increasing in GNP similar to those of *osckx8* and *osckx11* single‐gene mutants (Figure 4). However, only the *osckx3/8/11* mutant produced more branches of panicle than the WT, like *osckx8* and *osckx11* single‐gene mutants. And the seed setting rate of *osckx3/8/11* mutant was significantly reduced, which was the same as that of the *osckx11* mutant. Unlike *osckx8* single‐gene mutant, there were no differences of branch number between *osckx3/8* and WT. Moreover, there were no differences of 1,000‐grain weight and grain size between *osckx3/8* and WT (Figures 2 and 4). Based on these results, we might speculate that *OsCKX3* and *OsCKX8* probably were functionally complementary. Which resulted in a different phenotype of the *osckx3/8* double mutant and *osckx8* single mutant. *OsCKX3* might have partially restored the effect of *OsCKX8* on grain size and panicle development. On the other hand, the role of the *OsCKX11* in the *OsCKX3/8/11* clade was probably the most important, which might be related to the highest expression of *OsCKX11* in most tissues.

### 4.3 *OsCKX4/5/9* gene clade regulated the development of roots and shoots, and affected plant types such as plant height and tillers

It was well recognized that CTKs was associated with cell division, and elevated CTKs could promote the development of meristem tissue, shoot apical meristem (SAM), and root apical meristem (RAM) (Khandal et al., 2020; Ramireddy et al., 2018; Reid et al., 2016; Werner et al., 2010). Overexpression of *AtCKX* led to enhanced root growth, including greater lateral root density (Köllmer et al., 2014; Werner & Schmulling, 2009; Werner et al., 2003). The root system of cereals was significantly different from that of Arabidopsis. Overexpression and down‐regulation (RNAi and CRISPR‐Cas9 knockout) of *OsCKX4* led to greater and lesser crown root growth, respectively (Gao et al., 2014; Rong et al., 2022). It suggested that *OsCKX4* had a key role in the initiation of crown roots in rice. Duan's research found (Duan et al., 2019) that both a CRISPR‐Cas9 knockout mutant *ockx9* and overexpression of *OsCKX9* increased tiller number, reduced plant height and decreased panicle length and grain number. Furthermore, Rong discovered that the *osckx4/9* double mutant had an extremely higher tiller number compared with the two single mutants, as well the panicle size and the number of spikelet per panicle were markedly reduced (Rong et al., 2022).

In our studies, similar results were observed in *osckx4*, *osckx5*, and *osckx9* single‐gene mutants, which were grouped into the same clade. *osckx4*, *osckx5*, and *osckx9* single mutants all resulted in a clear decrease of plant height, whereas *osckx4* and *osckx9* were also accompanied by an increase in tiller number (Figure 2a–c). Loss of function of *OsCKX4* resulted in smaller grain sizes, lower 1,000‐grain weight and seed germination rate, and less and shorter crown roots (Figure 2h‐‐k). We did not observe changes in other agronomic traits in *osckx4*, *osckx5*, and *osckx9* single‐gene mutant. *osckx4/9* double mutant and *osckx4/5/9* triple mutant led to more significant reduction of plant height than that of single‐gene mutants and WT (Figure 4a and 4b; Supplemental Figure S4a). The leaves and stems size of *osckx4/9* and *osckx4/5/9* mutants were also smaller than that of the WT (data not shown). At the same time, *osckx4/9* and *osckx4/5/9* multiple mutants led to an extreme increase of tiller number, reaching three to five times that of the WT (Supplemental Figure S4b). Unlike the single‐gene mutants, the agronomic traits of *osckx4/9* and *osckx4/5/9* multiple mutants including the panicle size (length), the GNP, the primary and secondary branch number of panicle, the grain size (length and width), the 1,000‐grain weight, the seed germination rate, root length, and seedling height were all significantly affected and extremely lower than the WT (Figure 4; Supplemental Figure S4–S9). Based on above data, we found significant functional redundancy among the *OsCKX* members in the same clade, which worked synergistically to play a regulatory role in rice growth and development such as plant type, grain type, and grain size.

### 4.4 *OsCKXs* regulated leaf senescence and affected the grain quality

Previous studies had shown that CTK was involved in the regulation of leaf development and senescence, and high levels of CTK content delayed plant leaf senescence (Wu et al., 2021). Overexpression of different *AtCKX* genes led to a significant reduction in leaf size, but the aging of the leaves of the transgenic plants was not significantly affected (Hutchison et al., 2006; Werner et al., 2003). The CKX isoforms in the tomato (*Solanum lycopersicum* L.) leaf appeared to have distinct roles in differentially regulating CTK levels and indirectly influencing H_2_O_2_ accumulation, whereas H_2_O_2_ was involved in chlorophyll degradation (Cueno et al., 2012). *OsCKX11* is the most strongly expressing CKX gene family member in the root, leaf, and early rice panicles. And the expression level of *OsCKX11* was significantly higher than that of other *OsCKXs* members in the senescent leaves (Yamburenko et al., 2017; Zhang et al., 2021a). Zhang found that the chlorophyll in the leaves of *osckx11* knockout mutant was significantly higher than that of the WT in the rice mature stage and the aging stage. It elucidated that *OsCKX11* regulated the effects of leaf senescence by reducing the endogenous ABA levels (Zhang et al., 2021a). In our study, there was no significant difference between the mutants and WT, whether the *osckx11* single mutant or the *osckx3/8/11* multiple mutant. Interestingly, the leaves of the *osckx4/9* and *osckx4/5/9* mutants were significantly yellower than that of the WT, whereas the leaves of all the *OsCKXs* single‐gene mutants did not show this phenotype. How the *OsCKX4, OsCKX5*, and *OsCKX9* genes worked together to influence the chlorophyll content and leaf development remained to be further studied.

To investigate the effect of *OsCKXs* gene mutations on rice harvest timing, we assessed panicle senescence from the heading to full grain maturity stages. Although there were no significant differences in leaf senescence between *osckx* mutants and the WT, almost all mutants were moderately delayed in the panicle aging compared with the WT. In *osckx4*, *osckx7, osckx11* single‐gene mutants and *osckx3/8/11, osckx4/9*, and *osckx4/5/9* multi‐gene mutants, delayed panicle senescence might affect the grain grouting speed and reduced the seed setting rate. In addition, the extended harvest timing led to the mutants, which harvested at the same time as WT, having great differences in appearance and quality such as grain color, chalk rate, total starch content, and amylose content. In particular, the amylose content of all the *OsCKXs* mutants was significantly lower than that of WT (Figure 5; Supplemental Figure S10). Further research would be carried on whether there was an association between the *OsCKXs* genes and starch quality, and whether *OsCKXs* played a role in regulating amylose content and starch synthesis.

### 4.5 *OsCKXs* played multiple roles by regulating endogenous tZR and iP levels

Naturally occurring CTKs, which were N6‐substituted adenine derivatives, could be divided into two groups: those with an isoprenoid side chain and those with an aromatic one (Haberer & Kieber, 2002; Sakakibara, 2006; Werner & Schmulling, 2009; Werner et al., 2001). The isoprenoid CTKs were considered to be the predominant forms (Sakakibara, 2006), because the aromatics were not detected in cereals either maize leaves (Lacuesta et al., 2018) or rice (Kudo et al., 2010). Isoprenoid CTKs were primarily composed of iP (isopentenyl adenine), tZ (trans‐zeatin), cZ (cis‐zeatin), and DZ (dihydrozeatin), of which iP and tZ were considered the main active CTKs (Chen et al., 2021; Kudo et al., 2010; Sakakibara, 2006). The CTK levels were controlled through biosynthesis by IPT, destruction by CKX, and inactivation through glucosylation by cytokinin glucosyl transferases (CGTs). The CKXs reduced the level of active CTKs by irreversibly cutting the free radicals and ribose forms of CTKs on the N6 side chain. Both tZ and iP were cleaved by CKXs, but DZ and synthetic CTKs were resistant to CKXs cleavage (Chen et al., 2021; Liu et al., 2020).

The analysis of endogenous hormone content in the leaves of different mutants showed that knockout of different *OsCKXs* would reduce the activity of CKX and cause the rise of different CTKs in rice. The tZR and iP contents in *osckx11, osckx4/9*, *osckx4/5/9, osckx3/8/11* mutants were significantly increased compared with the WT (Figure 6). Conversely, the tZR content in *osckx5, osckx6*, and *osckx7* knockout mutants decreased significantly (Figure 6). The results indicated that the *OsCKXs* gene might be involved in and played an important role in regulating the degradation of tZR and iP in rice leaves. These results provided a basis for further research on the role of the *OsCKX* gene family in the metabolic network of CTKs, as well as the role of metabolic balance between different endogenous hormones.

## AUTHOR CONTRIBUTIONS

**Xuelian Zheng**: Formal analysis; Funding acquisition; Project administration; Resources; Supervision; Validation; Writing – original draft; Writing – review & editing. **Shuting Zhang**: Data curation; Formal analysis; Methodology; Validation. **Yanling Liang**: Data curation; Formal analysis; Investigation; Validation. **Rui Zhang**: Formal analysis; Software; Validation; Visualization. **Li Liu**: Investigation; Software; Validation. **Pengchen Qin**: Data curation; Formal analysis; Investigation. **Zhe Zhang**: Software; Validation; Visualization. **Yan Wang**: Data curation; Investigation; Resources. **Jianping Zhou**: Funding acquisition; Resources. **Xu Tang**: Funding acquisition; Methodology; Resources. **Yong Zhang**: Conceptualization; Funding acquisition; Project administration; Resources; Supervision; Writing – review & editing.

## CONFLICT OF INTEREST

The authors declare no competing interests.

## Supporting information

Supplemental Figure S1. Chromosomal localization and protein sequence alignment of 11 *OsCKX* genes. The FAD‐binding domain and cytokinin‐binding domain were showed above lines.
Supplemental Figure S2. DNA sequencing reveals the genotype of *OsCKX* single‐gene mutant plants. The PAM sequence is in red and the protospacer sequence is in blue.
Supplemental Figure S3. DNA sequencing reveals the genotype of *OsCKX* multiple‐gene mutant plants. The PAM sequence is in red and the protospacer sequence is in blue.
Supplemental Figure S4. Plant height, tiller number and seed setting rate of all *OsCKX* single‐gene mutants and multiple‐gene mutants. Error bars represent standard deviations of three biological replicates. Data are means ± SD, *n* = 3. *p < .05; **p < .01 (Two‐tailed Student's *t* tests).
Supplemental Figure S5. Panicle length and grain number per panicle of all *OsCKX* single‐gene mutants and multiple‐gene mutants. Data are means ± SD, *n* = 3. *p < .05; **p < .01 (Two‐tailed Student's *t* tests).
Supplementary Figure S6. The primary branch number per panicle and secondary branch number per panicle of all *OsCKX* single‐gene mutants and multiple‐gene mutants. Data are means ± SD, *n* = 3. *p < .05; **p < .01 (Two‐tailed Student's *t* tests).
Supplemental Figure S7. Grain size of *OsCKX* single‐gene mutants and multiple‐gene mutants without difference compared with wild‐type.
Supplemental Figure S8. 1,000‐grain weight, grain length and grain width of all *OsCKX* single‐gene mutants and multiple‐gene mutants grown in the greenhouse. Error bars represent standard deviations of three biological replicates. Data are means ± SD, *n* = 200 for 1,000‐grain weight, and *n* = 30 for the others. *p < .05; **p < .01 (Two‐tailed Student's *t* tests).
Supplemental Figure S9. Seed germination rate (*n* = 30), shoot and root length of all *OsCKX* single‐gene mutants and multiple‐gene mutants. Data are means ± SD, n = 10. *p < .05; **p < .01 (Two‐tailed Student's *t* tests).
Supplemental Figure S10. Seed chalky rate and total starch content of all *OsCKX* single‐gene mutants and multiple‐gene mutants. Bar = 1 cm. Error bars represent standard deviations of three biological replicates. Data are means ± SD, *p < .05; **p < .01 (Two‐tailed Student's *t* tests).

Supplemental Table S1. Heredity test at different targeted sites in T1 generation.
Supplemental Table S2. Constructs used in this study.
Supplemental Table S3. Oligos used in this study.

## Data Availability

All data generated or analyzed during this study are included in this published article (and its supplementary information files).
