# Supplementary material for: Loss‐function mutants of OsCKX gene family based on CRISPR‐Cas systems revealed their diversified roles in rice
Source: Plant Genome. 2023 Jan 20;16(2):e20283. doi: 10.1002/tpg2.20283 (PMC12807126; doi:10.1002/tpg2.20283)
Supplement: Supplementary file 1 [file TPG2-16-e20283-s001.pdf]

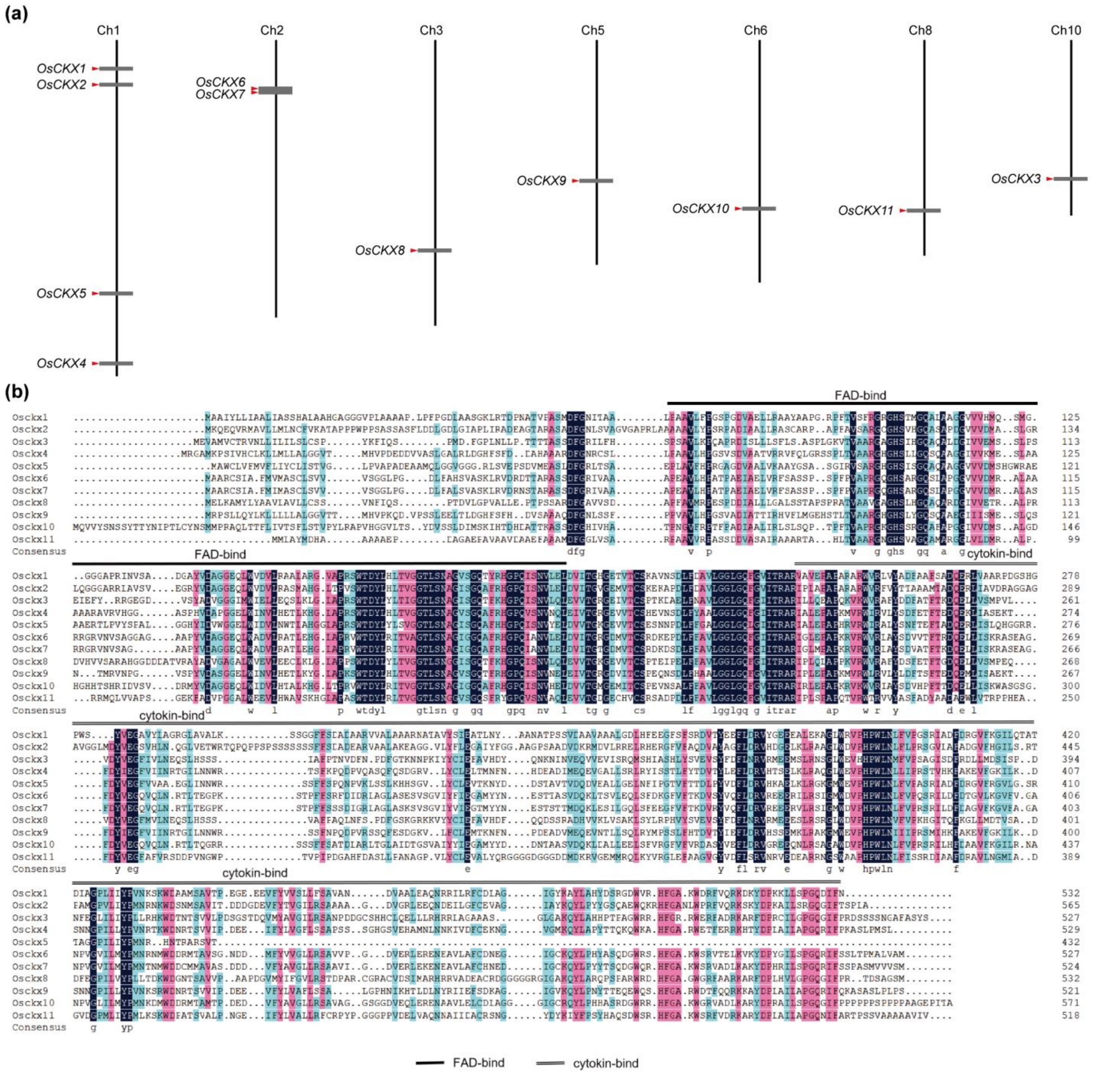

Supplementary Figure S1. The Chromosomal localization (a) and protein sequence alignment (b) of 11 *OsCKX* genes. The FAD-binding domain and cytokinin-binding domain were showed above lines.

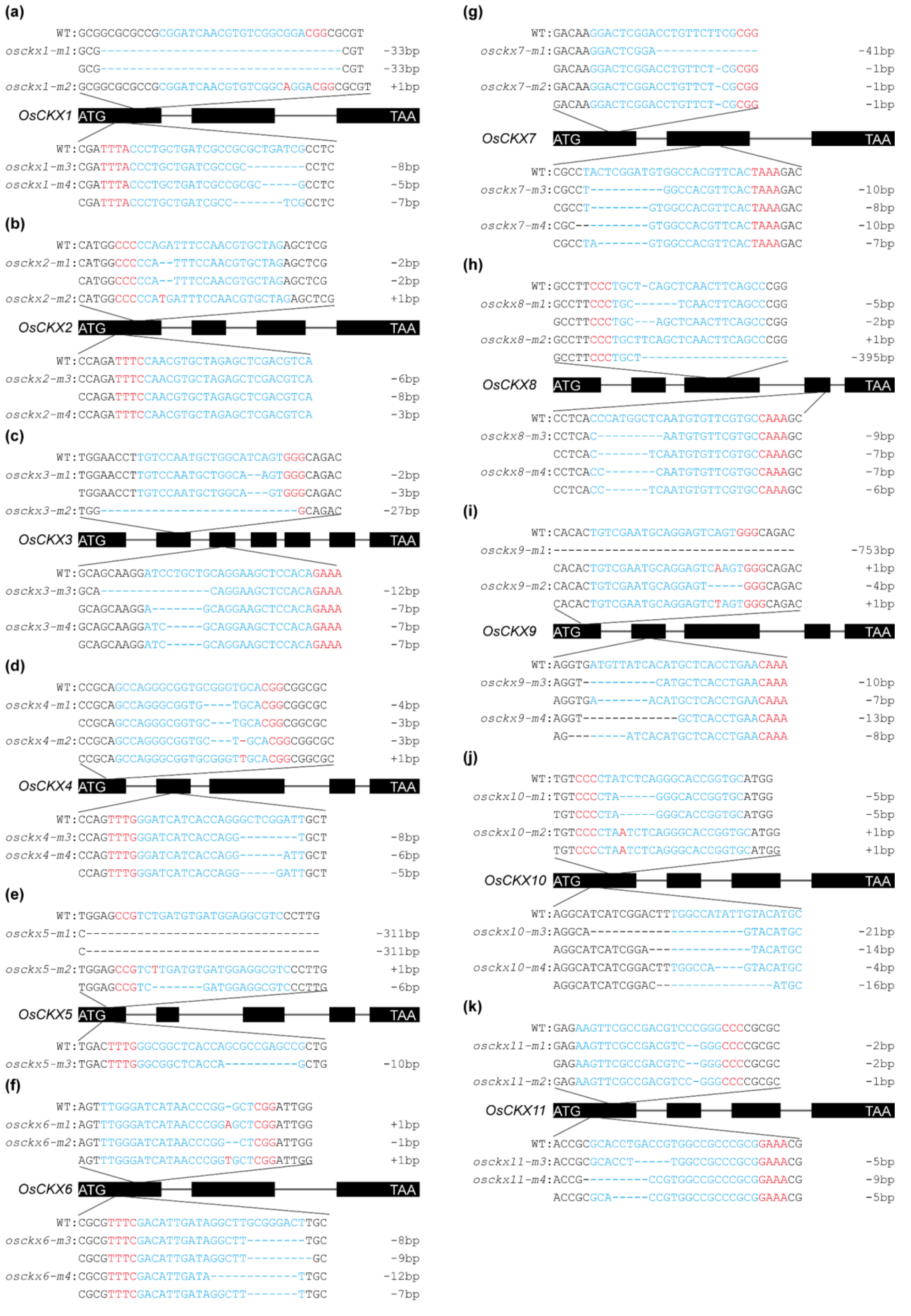

Supplementary Figure S2. DNA sequencing reveals the genotype of *OsCKX* single-gene mutant plants. The PAM sequence is in red and the protospacer sequence is in blue.

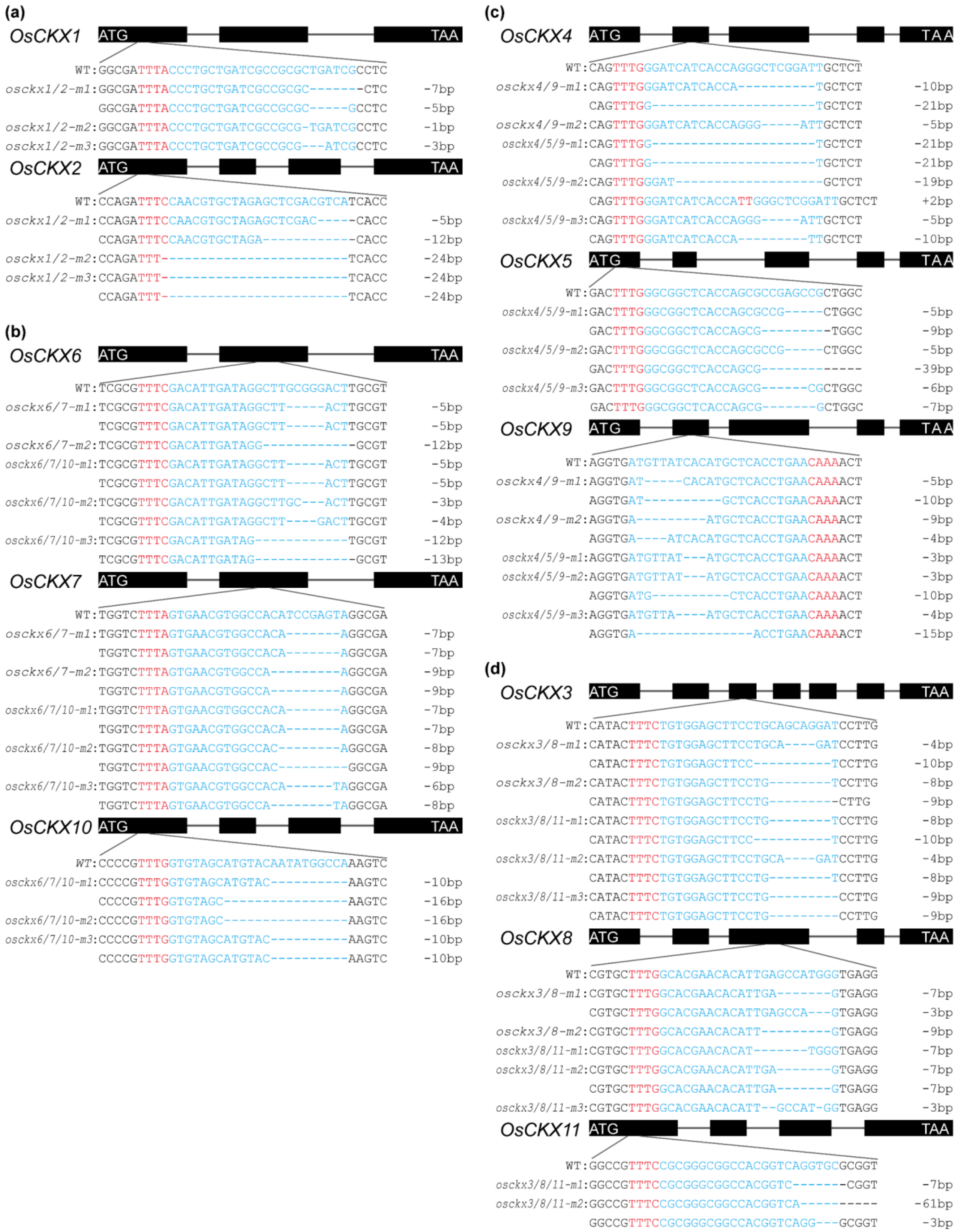

Supplementary Figure S3. DNA sequencing reveals the genotype of *OsCKX* multiple-gene mutant plants. The PAM sequence is in red and the protospacer sequence is in blue.

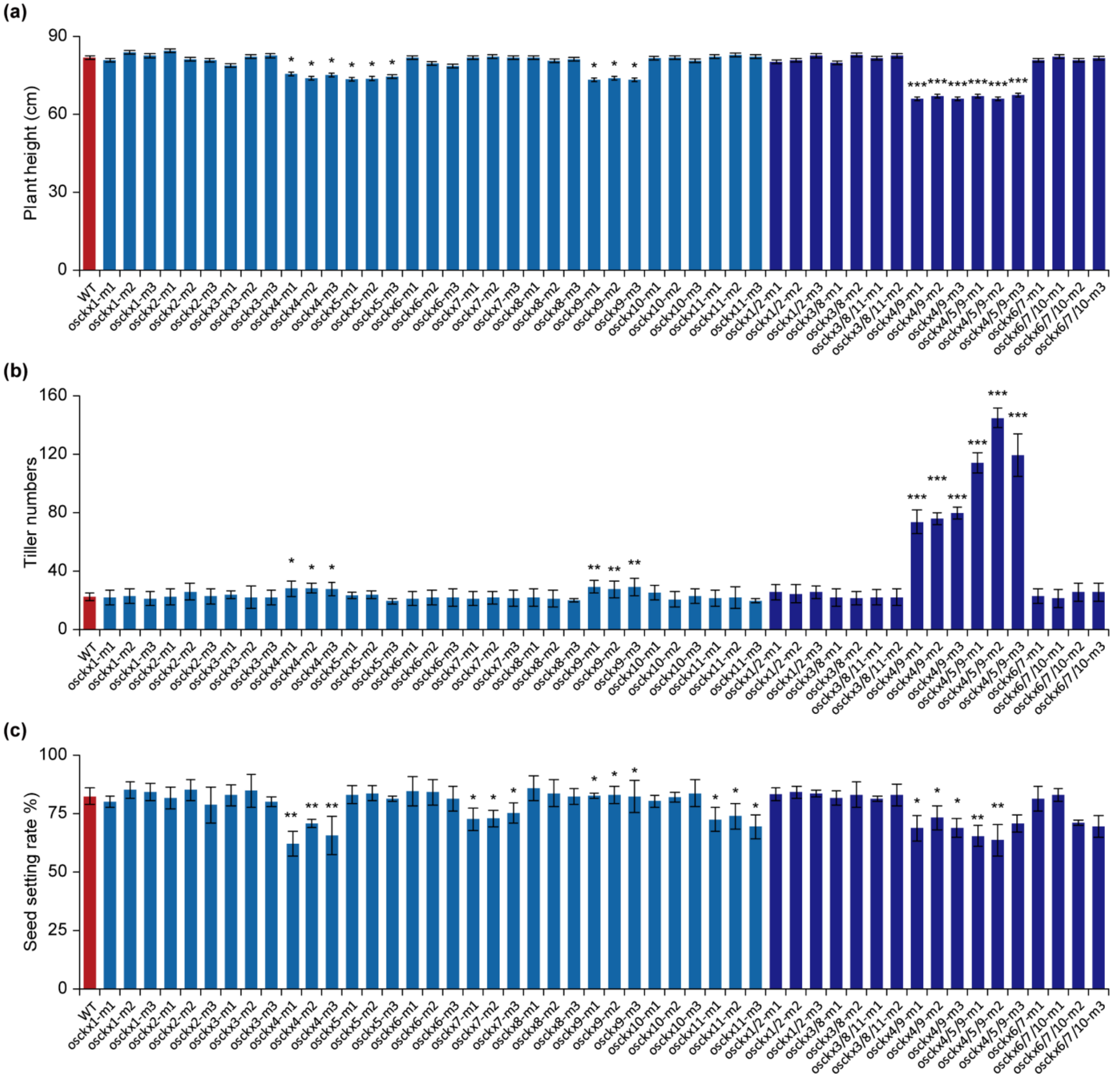

Supplementary Figure S4. Plant height, tiller number and seed setting rate of all *OsCKX* single-gene mutants and multiple-gene mutants. Error bars represent standard deviations of three biological replicates. Data are means ± SD, n = 3. *, $P < 0.05$; **, $P < 0.01$; ***, $P < 0.001$ (Two-tailed Student's t-tests).

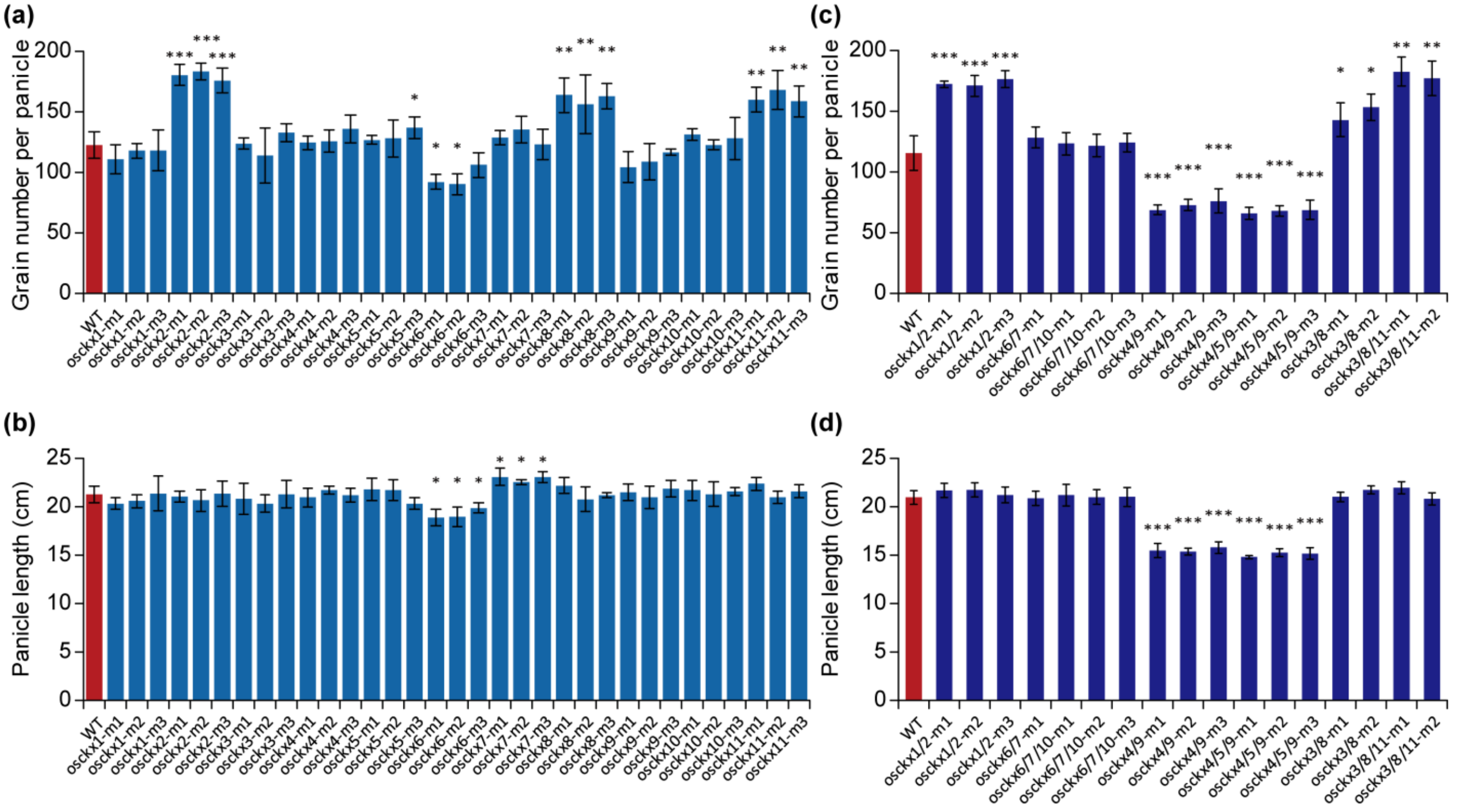

Supplementary Figure S5. Panicle length and grain number per panicle of all *OsCKX* single-gene mutants and multiple-gene mutants. Data are means ± SD, n = 3. *, $P < 0.05$; **, $P < 0.01$; ***, $P < 0.001$ (Two-tailed Student's t-tests).

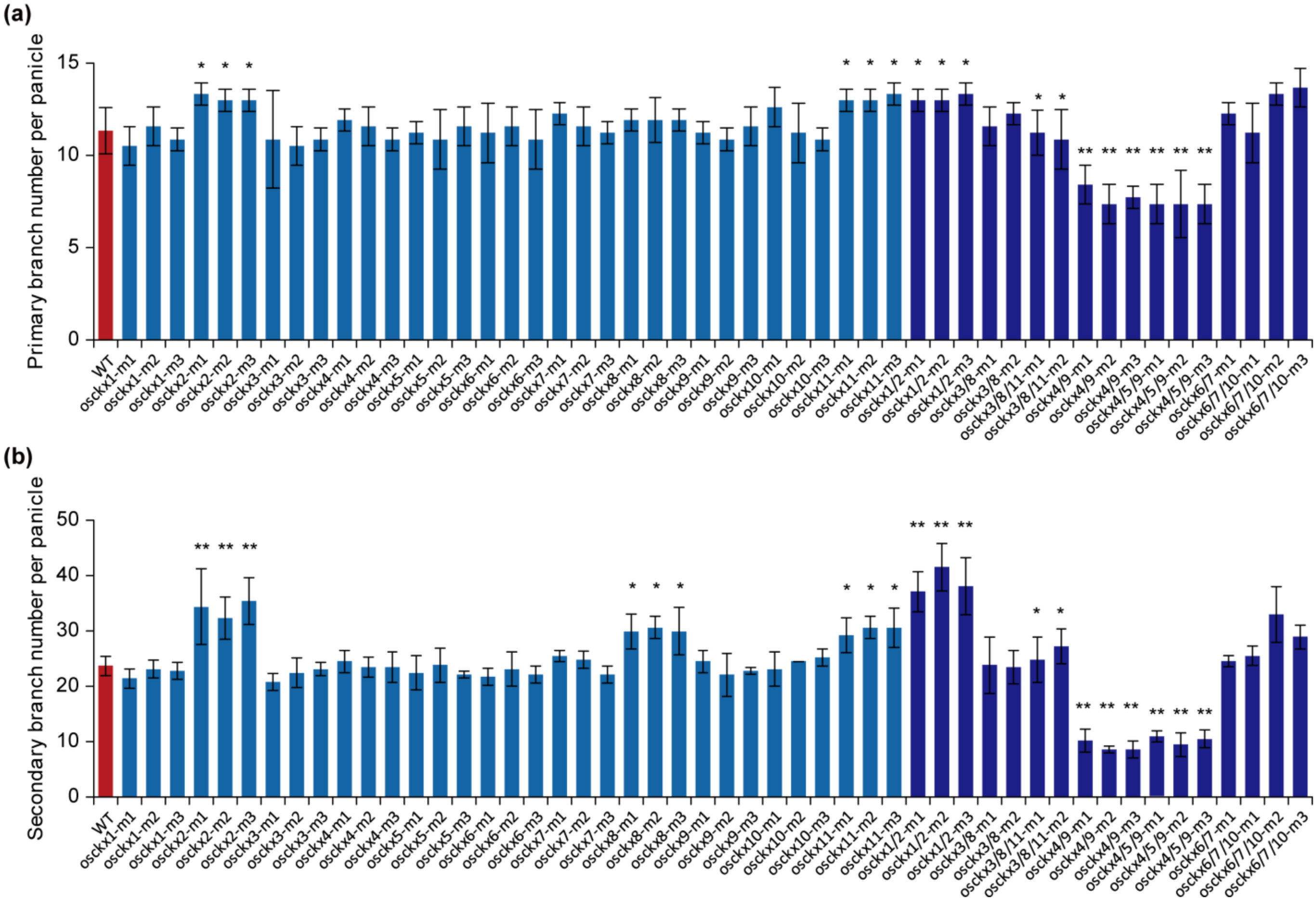

Supplementary Figure S6. The primary branch number per panicle and secondary branch number per panicle of all *OsCKX* single-gene mutants and multiple-gene mutants. Data are means ± SD, n = 3. *, P < 0.05; **, P < 0.01 (Two-tailed Student's t-tests).

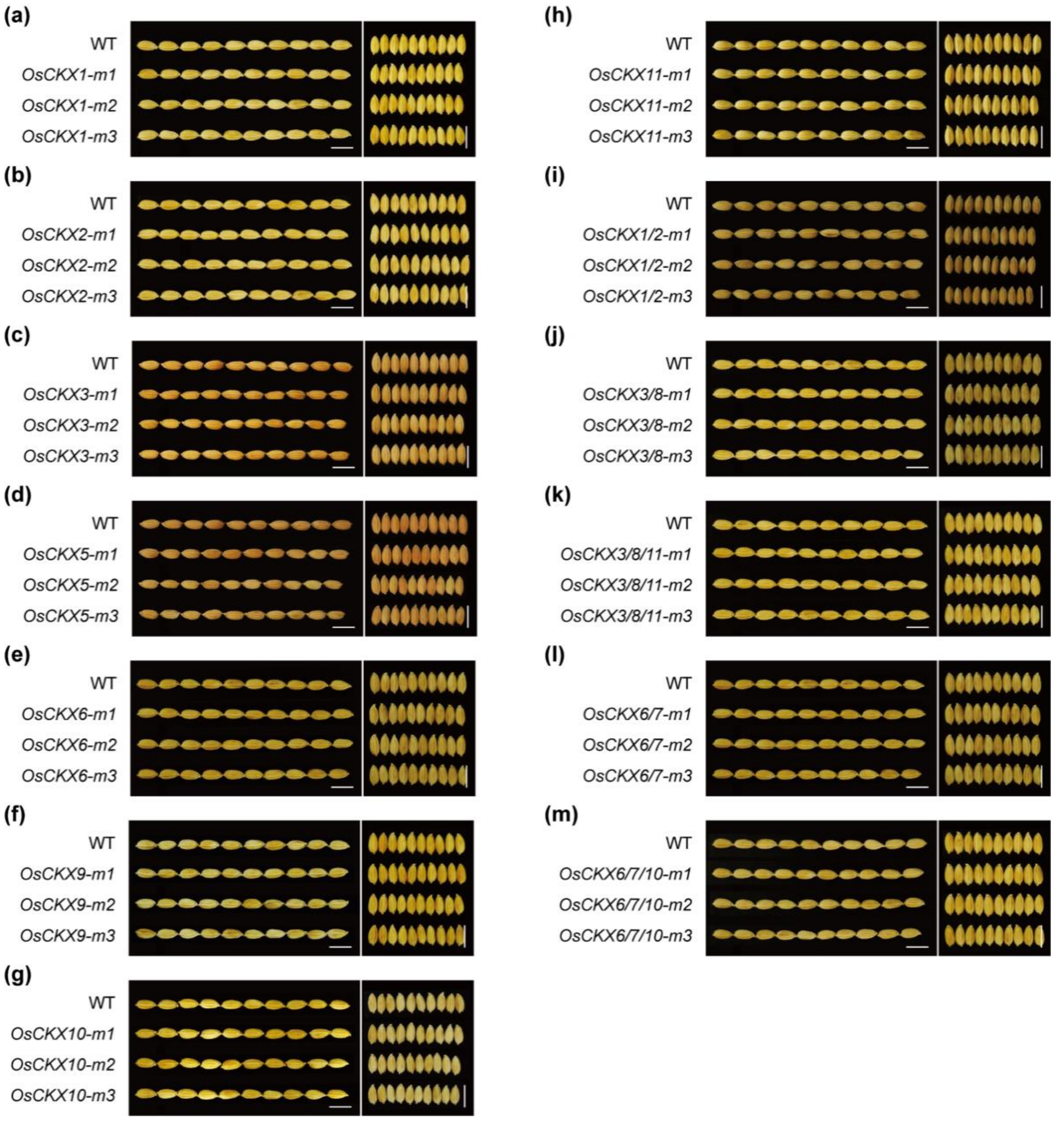

Supplementary Figure S7. Grain size of *OsCKX* single-gene mutants and multiple-gene mutants without difference compared to wild-type.

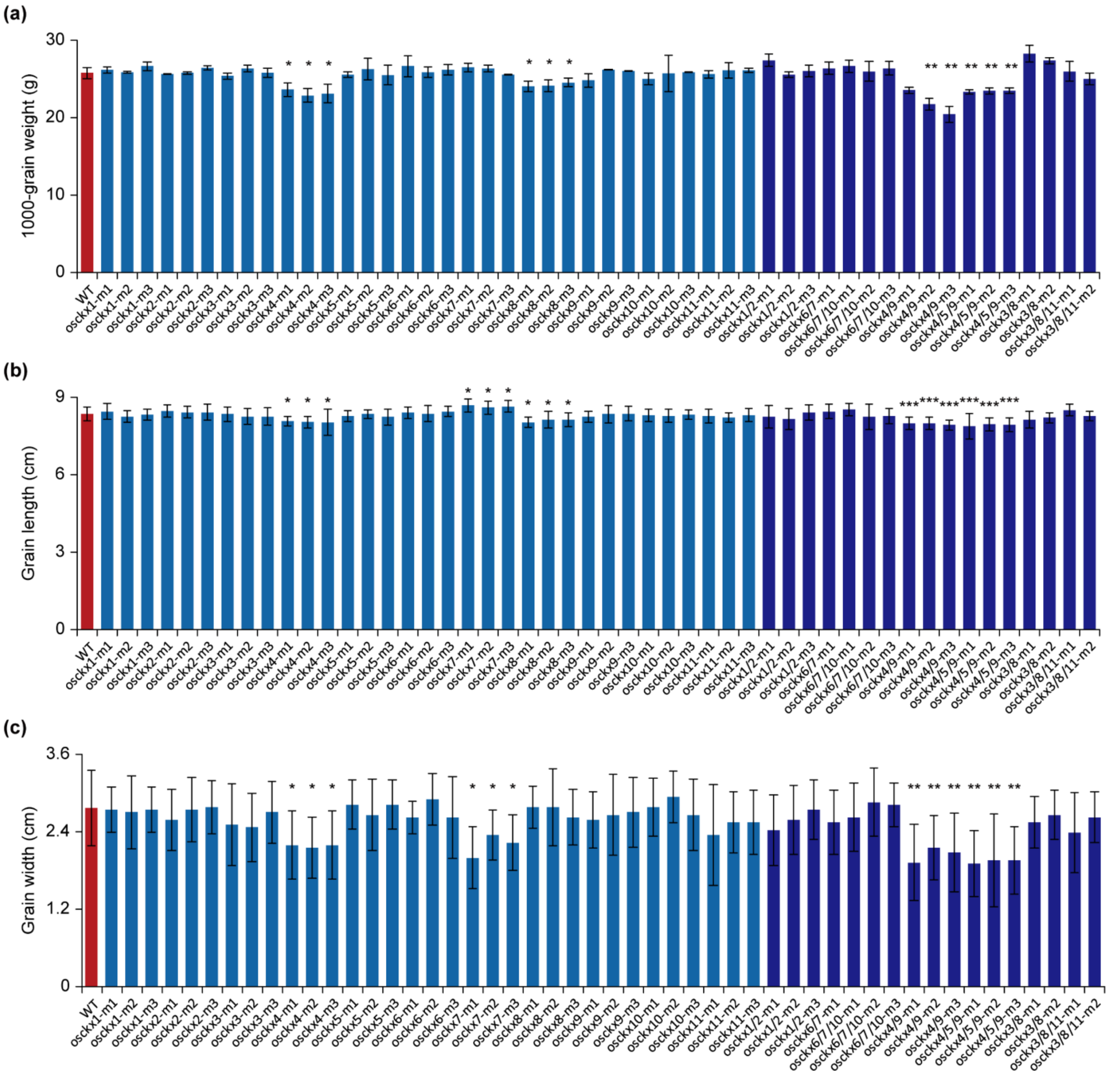

Supplementary Figure S8. 1000-grain weight, grain length and grain width of all *OsCKX* single-gene mutants and multiple-gene mutants grown in the greenhouse. Error bars represent standard deviations of three biological replicates. Data are means ± SD, n=200 for 1000-grain weight, and n = 30 for the others. *, P < 0.05; **, P < 0.01 ; ***, P < 0.001 (Two-tailed Student's t-tests).

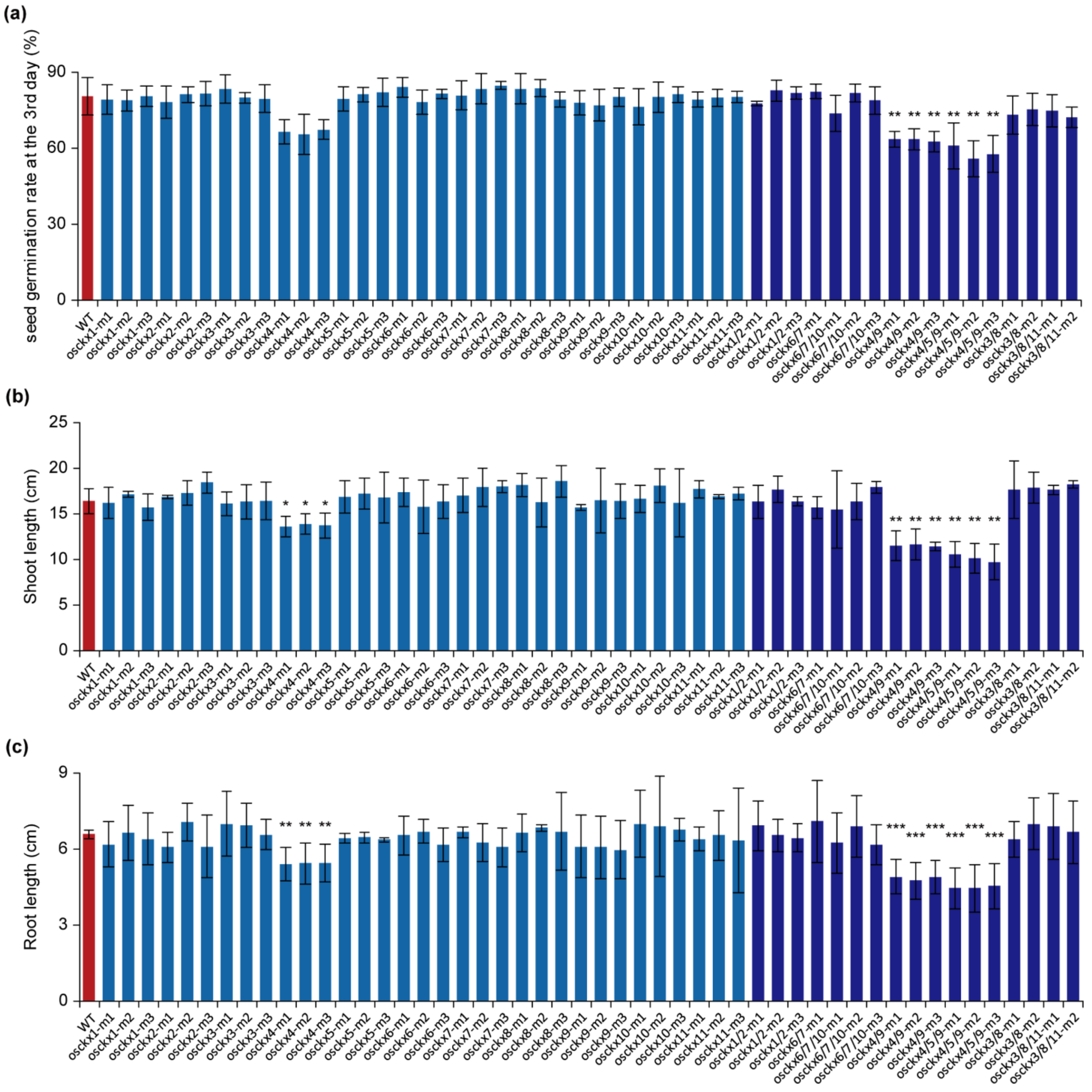

Supplementary Figure S9. Seed germination rate (n=30), shoot and root length of all *OsCKX* single-gene mutants and multiple-gene mutants. Data are means ± SD, n = 10. *, $P < 0.05$; **, $P < 0.01$; ***, $P < 0.001$ (Two-tailed Student's t-tests).

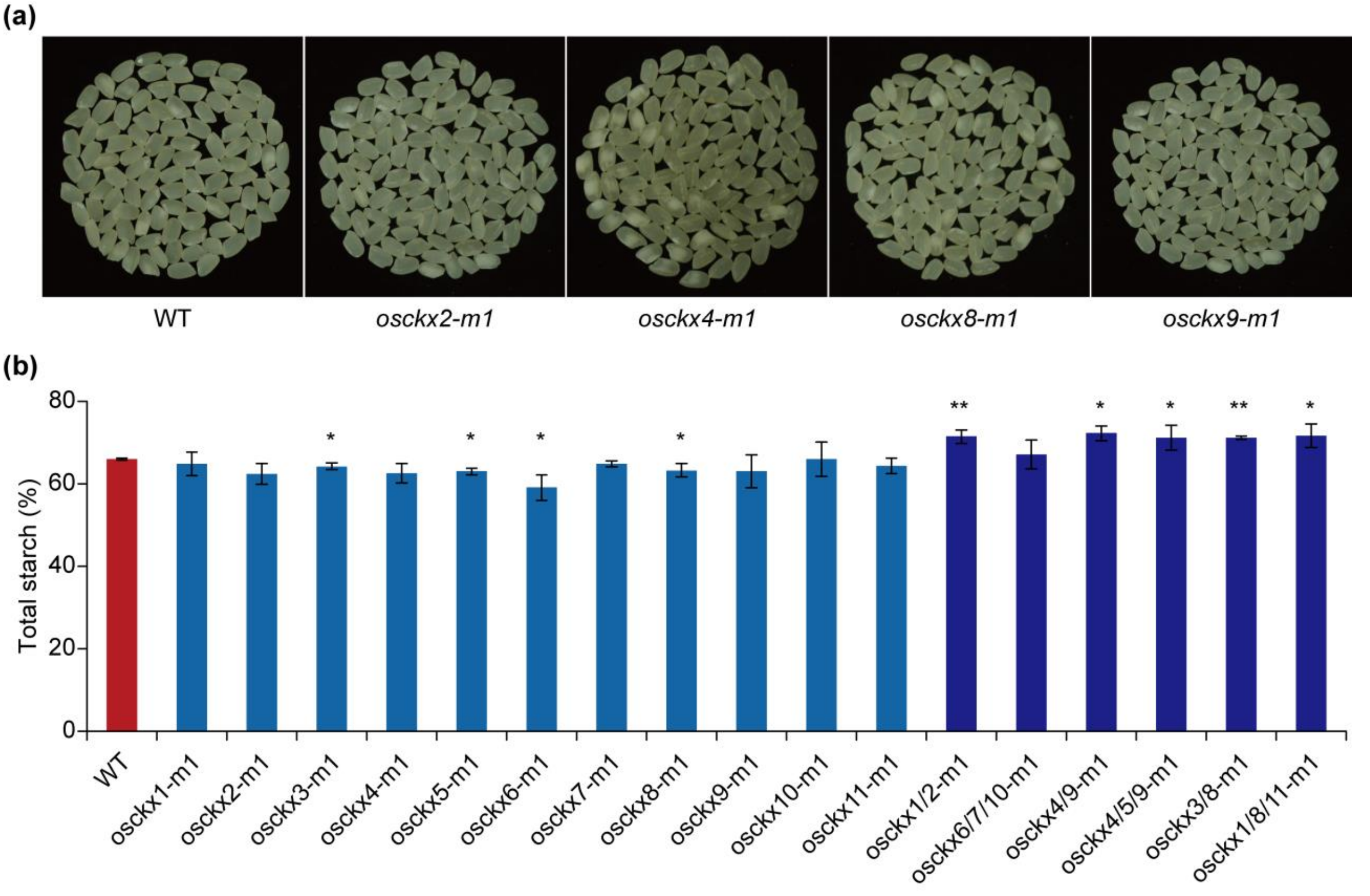

Supplementary Figure S10. **Seed chalky rate and total starch content of all *OsCKX* single-gene mutants and multiple-gene mutants.** Bar=1cm. Error bars represent standard deviations of three biological replicates. Data are means SD, *, P < 0.05; **, P < 0.01 (Two-tailed Student's t-tests).
